# Arsenic Trioxide Enhances the NK Cell Cytotoxicity Against Acute Promyelocytic Leukemia While Simultaneously Inhibiting Its Bio-Genesis

**DOI:** 10.3389/fimmu.2018.01357

**Published:** 2018-06-14

**Authors:** Ansu Abu Alex, Saravanan Ganesan, Hamenth Kumar Palani, Nithya Balasundaram, Sachin David, Kavitha M. Lakshmi, Uday P. Kulkarni, P. N. Nisham, Anu Korula, Anup J. Devasia, Nancy Beryl Janet, Aby Abraham, Alok Srivastava, Biju George, Rose Ann Padua, Christine Chomienne, Poonkuzhali Balasubramanian, Vikram Mathews

**Affiliations:** ^1^Department of Hematology, Christian Medical College, Vellore, India; ^2^UMR-S1131, Hôpital Saint Louis, Paris, France; ^3^Institut Universitaire d’Hématologie, Universite Paris Diderot, Paris, France

**Keywords:** natural killer cells, arsenic trioxide, immune response, acute promyelocytic leukemia, APL mouse model, NK cellular therapy

## Abstract

Natural killer cells (NK) contribute significantly to eradication of cancer cells, and there is increased interest in strategies to enhance it’s efficacy. Therapeutic agents used in the treatment of cancer can impact the immune system in a quantitative and qualitative manner. In this study, we evaluated the impact of arsenic trioxide (ATO) used in the management of acute promyelocytic leukemia (APL) on NK cell reconstitution and function. In patients with APL treated with single agent ATO, there was a significant delay in the reconstitution of circulating NK cells to reach median normal levels from the time of diagnosis (655 days for NK cells vs 145 and 265 days for T cells and B cells, respectively). *In vitro* experiments demonstrated that ATO significantly reduced the CD34 hematopoietic stem cell (HSC) differentiation to NK cells. Additional experimental data demonstrate that CD34^+^ sorted cells when exposed to ATO lead to a significant decrease in the expression of *IKZF2*, ETS1, and TOX transcription factors involved in NK cell differentiation and maturation. In contrast, exposure of NK cells and leukemic cells to low doses of ATO modulates NK cell receptors and malignant cell ligand profile in a direction that enhances NK cell mediated cytolytic activity. We have demonstrated that NK cytolytic activity toward NB4 cell line when exposed to ATO was significantly higher when compared with controls. We also validated this beneficial effect in a mouse model of APL were the median survival with ATO alone and ATO + NK was 44 days (range: 33–46) vs 54 days (range: 52–75). In conclusion, ATO has a differential quantitative and qualitative effect on NK cell activity. This information can potentially be exploited in the management of leukemia.

## Introduction

There is increased evidence that the immune system plays a role in the prevention of cancer and also in the maintenance of durable remission post chemotherapy (1). Cells of both innate and adaptive arms of the immune system contribute to immune surveillance (2), and there are mechanisms by which malignant cells can escape from such surveillance (3). Anti-cancer drugs can be lymphodepleting, immunostimulatory, or both (4, 5), and they also play a pivotal role in mediating anti-leukemic immune response (6, 7). Sensitization of malignant cells using chemotherapeutic agents helps in eliciting productive immune responses (8, 9).

Natural killer cells (NK) or innate lymphoid cells (ILCs) are a part of the innate immune system and are involved in the surveillance against malignant cells (10). Activated NK cells have been known to be effective in killing the tumor targets (11). The clinical efficacy of NK cell immunotherapy is well known in hematological malignancies (12). Various agents, such as all trans-retinoic acid and sodium valproate, were evaluated for their ability to induce NK ligands on leukemic cells and augmenting immune mediated anti-leukemic effect (13). Upregulation of functional activating receptors on NK cells by interleukin 15 (14), nicotinamide (15), or lenalidomide (16) to improve NK cell cytotoxicity has also been reported. Additional studies have shown the role of killer immunoglobulin-like receptor (KIR) gene haplotype as a predictor of disease outcome (17).

Acute promyelocytic leukemiaAPL is a subtype of acute myeloid leukemia (AML) characterized with distinct molecular and clinical features, and majority of cases characterized by the fusion between retinoic acid receptor alpha gene (RARα) on chromosome 17 and the partner gene promyelocytic leukemia (PML) on chromosome 15 as a result of reciprocal translocation t(15;17)(q24;q21), leading to the expression of a novel PML-RARα oncoprotein (18). Arsenic trioxide (ATO), as a single agent, is effective in the management of newly diagnosed cases of acute promyelocytic leukemia (APL). ATOArsenic trioxide, in a dose-dependent manner, exerts its therapeutic effect by promoting degradation of the oncoprotein that drives the growth of APL cells (19). Despite its efficacy in the treatment of APL, relapses occurs in 5–30% of cases mostly within the high-risk subset (20). It has been demonstrated that immune response is important in sustaining long-term molecular remission in a transplantable mouse model of APL (21, 22). There is are significant data which address the mechanisms of action of ATO on malignant promyelocytes (19, 23) and our own data looking at the extrinsic factors causing resistance to ATO (24), but limited data is are available on its effect on the innate and adaptive immune system. A few studies have shown the immunomodulatory property of ATO by up-regulating the NK ligands on tumor cells thereby increasing the susceptibility of cancer cells to NK cells (25).

Immune reconstitution following cessation of chemotherapy is one of the factors that have a potential impact on disease recurrence (26). Immune reconstitution has been studied extensively in hematological malignancies in context with hematopoietic stem cell transplantation (HSCT) in Ref. (27). NK cells were known to reconstitute rapidly post-transplant (28) while certain studies have shown impaired NK cell numbers and function (29, 30) post chemotherapy. There are limited data concerning long-term recovery of the immune subsets post chemotherapy in leukemia, and there are no data on the effect of ATO on immune reconstitution. Hence in this study we evaluated, the impact of ATO on the NK cell function and recovery and role of NK cellular therapy in combination with ATO.

## Materials and Methods

### Cell Lines and Primary Cells

**Figure 1:**
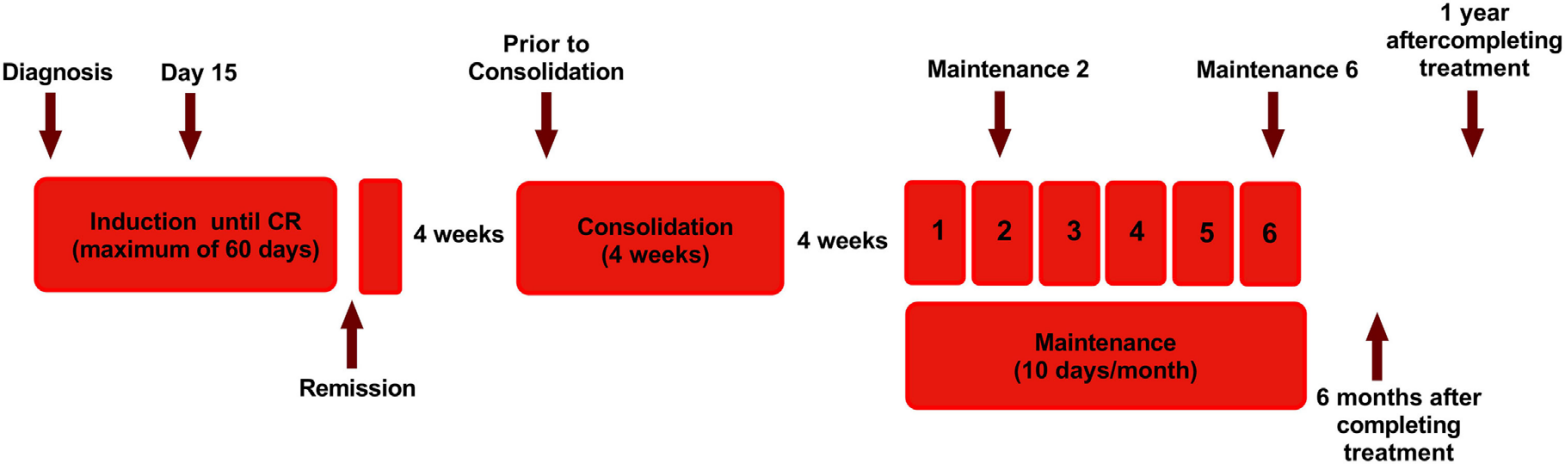
Diagram showing the treatment protocol followed in our institution for newly diagnosed acute promyelocytic leukemia and time points of sample collection for this study. Arsenic trioxide (ATO) was administered intravenously at a dose of 10 mg for adults and 0.15 mg/kg for pediatric patients. Single-agent ATO was administered until complete hematologic remission (CR) for a maximum of 60 days. Following a 4-week interval, ATO was administered for another 4 weeks as a consolidation course, for those in CR. Again after a 4-week interval, for those continuing to remain in CR, single agent ATO was administered 10 days a month for 6 months (31). The arrow marks represent the time points in which samples were collected.

The human APL cell lines used in this study were NB4 (kind gift from Dr. Harry Iland, RPAH, Sydney, NSW, Australia with permission from Dr. Michel Lanotte), all-trans retinoic acid resistant cell line UF1 (kind gift from Dr. Christine Chomienne, Hôpital Saint-Louis, Paris) and an in-house generated ATO-resistant cell line NB4-EVAsR1 (detailed methodology in Supplementary Methods S1 in Supplementary Material). The myeloid cell lines K562, HL60, and U937, the lymphoid cell lines Jurkat E6.1 and SUP-B15, and NK cell line NK92MI were obtained from American Type Culture Collection (ATCC, Rockville, MD, USA). The cell lines were cultured at 37°C in a humidified atmosphere containing 5% CO_2_ and were characterized phenotypically by flow cytometry and free of mycoplasma contamination (Universal Mycoplasma detection Kit, ATCC). Patients newly diagnosed with APL from March 2010 to May 2015 (*n* = 112) were prospectively enrolled in this study after getting written and informed consent. The patients were treated with single agent ATO as has been previously reported by us (31, 32) (detailed treatment protocol in Supplementary Methods S2). Peripheral blood samples were collected at different treatment time points as depicted in Figure 1. Samples were also collected from patients who have completed treatment for more than 2 years. The study was approved by the institutional review board, Christian Medical College, Vellore (IRB Min no: 7081 dated 17.02.2010) and have been performed in accordance with the ethical standards as laid down in the 1964 Declaration of Helsinki and its later amendments or comparable ethical standards.

### *In Vitro* ATO Cytotoxicity Assay

The *in vitro* sensitivity of the malignant cell lines toward ATO was determined at 48 h using MTT assay (Biotium, Inc., CA, USA) as described previously (24). All experiments were done in triplicates and the half-maximal inhibitory concentration (IC50) values were generated using Graph Pad Prism V6 software (La Jolla, CA, USA).

### NK Cell Cytotoxicity Assay

The cytotoxic activity of NK cell line NK92MI against malignant myeloid (K562, U937, HL60, UF1, NB4, and NB4-EVAsR1) and lymphoid cell lines (Jurkat E6.1, SUP-B15) was assessed using the CFSE/7AAD cytotoxicity assay kit (Cell Technology, Mountain view, CA, USA). Briefly the effector cells (NK cells) and CFSE (carboxyfluorescein diacetate succinimidyl ester) stained target cells (1 × 10^5^ leukemic cells) were cocultured in different ratios 1:1, 2:1, 5:1, 10:1 in a 24-well plate with 500 µl 10% RPMI media. At the end of incubation at 37°C for 5 h, the cells were washed, and 2.5 µl of 7AAD was added to the samples and acquired in FACS Calibur (Becton Dickinson, Mansfield, MA, USA). The percentage of cytotoxicity was calculated, and the spontaneous death of the target cells was subtracted as background control. In a parallel set of experiments, the leukemic cell lines or NK cell line were exposed to 1 µM ATO overnight for 12 h and cytotoxicity was measured as described above.

### NK Cell Proliferation Assay

NK92MI (1 × 10^6^ cells) were left untreated or treated with 1 µM ATO and seeded in 24-well plates in 500 µl minimal essential medium (MEM) supplemented with 10% FBS and checked for the proliferation. The intensity of CFSE was measured by flow cytometry using BD FACS Calibur at FL1 channel at 24, 48, and 72 h respectively.

### NK Cell Degranulation Assay

NK92MI (5 × 10^5^ cells/well) was plated in 96-well U-bottom plates at in the presence of CD107a (BD Pharmingen, San Diego, CA, USA) and was resuspended in 200 µl 10% RPMI media. Degranulation was induced by adding the leukemic target cells (5 × 10^5^ per well, effector/target [E:T] ratio 2:1). At the end of incubation at 37°C for 5 h, CD56 was added and incubated for 20 min followed by PBS wash and were acquired in FACS Calibur (Becton Dickinson, Mansfield, MA, USA). The percentage of CD107a^+^CD56^+^ cells was measured. In another set of experiments, the target cells were treated with 1 µM ATO for 12 h, and NK cells were measured for degranulation. Similarly in APL patients who were on maintenance therapy with ATO, CD107a expression was measured by gating on CD56^+^CD3^−^ cells with and without adding target cells (NB4) (*n* = 9) and compared with healthy controls (*n* = 7).

### Immunophenotyping Studies

To check for the expression of activating and inhibitory receptors on NK92MI cell line and NK ligands on leukemic cells, 1 × 10^6^ cells were seeded in a 24-well plate and were left untreated or treated with 1 and 2 µM ATO for 6 and 24 h. The cells were then washed and stained with antibodies to NK cell receptors, and ligands along with the respective isotypic control antibodies (details of antibodies used are provided in Supplementary Methods S3) followed by incubation in the dark for 20 min. Unbound antibodies were removed by washing with phosphate buffer saline, and flow cytometric analysis was carried out in BD FACS Calibur (Becton Dickinson, Mansfield, MA, USA). The data were analyzed using BD CellQuest Pro software and plotted as histograms.

For immune reconstitution studies, peripheral blood samples were collected from newly diagnosed APL patients and stained with monoclonal antibodies and analyzed for the T cell subsets, B cells, NK cell subsets, and dendritic cells (Supplementary Methods S3). Following incubation with antibodies, a standard NH4Cl whole-blood lysing technique was done, and the washed cells were acquired and analyzed. Cell surface analysis was performed with a BD FACS Calibur flow cytometer (BD Biosciences, San Jose, CA, USA) using Cell quest pro software and absolute counts were calculated.

### Genotyping of Human KIR Genes

Genomic DNA was extracted from the bone marrow or peripheral blood samples from APL patients by standard protocols, and KIR genotyping was performed by sequence-specific primers (SSP-PCR) using KIR typing kit (Miltenyi Biotech, Bergisch Gladbach, Germany), and the presence or absence of 15 human KIR genes plus two pseudo genes were analyzed.

### NK Cell Therapy in APL Mouse Model

FVB/N mice were obtained from Jackson Laboratory (Bar Harbor, ME, USA). Mice at 6–8 weeks of age were used in all the experiments. The animal study design and euthanasia protocols were approved by the institutional animal ethics committee, Christian Medical College, Vellore (IAEC approval number 2/2012). Cells from the spleen of MRP8-PML-RARa transgenic FVB/N mice (33) were used to create APL transplantable model. APL blast cells were then harvested and cryopreserved (a kind gift from Dr. Christine Chomienne, Hôpital Saint-Louis with the permission from Dr. Scott Kogan and Dr. Michael Bishop). APL cells (5 × 10^4^ cells/mouse) were injected intravenously *via* the tail vein into genetically compatible FVB/N recipients, without conditioning with either radiation or chemotherapy. Leukemic mice were then divided into following groups: ATO alone, NK cells alone, ATO + NK, ATO + IL-15, ATO + NK + IL-15, and placebo group. ATO was given intraperitoneally at the concentration of 5 µg/g of mice starting on day 7 post injection of malignant cells and continued for 28 days. NK cells were isolated from the spleen of normal FVB/N and a total of 5 × 10^5^ NK cells were injected intravenously *via* the tail vein for 3 doses with 10 days interval. 100 ng of recombinant mouse IL-15 was given intraperitoneally for a total of 5 doses with 5 days interval and survival was monitored (details of the methodology are provided in Supplementary Methods S4).

### Stem Cell-Derived NK Cell Differentiation

CD34^+^ cells were sorted from umbilical cord blood samples obtained after getting written and informed consent (approved by institutional review board (Ethics Committee) of Christian Medical College, Vellore, EC min no. IRB (EC) 16/08/2006) using EasySep Human CD34 positive selection Kit (Stem cell Technologies, Vancouver, Canada) and was cultured in NK differentiation medium (10%RPMI + 10 ng SCF + 30 ng FLT3 + 50 ng IL-15) (Supplementary Methods S5). They were assessed for NK differentiation on day 8 and day 14 with or without exposure to 0.5 µM ATO by flow cytometry.

### Quantitative Real-Time PCR (RQ-PCR) for NK Cell Transcription Factors

RNA was extracted from CD34 cells in culture with or without exposure to 0.5 µM ATO on day 0 and day 14. The expression levels of NK transcription factors EOMES, IKZF2, PRDM1, KLF4, ETS1, TOX, and TBX21 were determined based on TaqMan^®^ Gene Expression Assays (Supplementary Methods S6).

### Statistical Analysis

Data were represented as mean of values ± SD or as median values with range as indicated in the figure legends. Student’s *t*-test or Mann–Whitney *U* test was used to statistically compare the continuous variables. For reconstitution, graphs values were plotted as median with interquartile ranges. The relationships of clinical features to outcome were analyzed by Cox proportional hazard model. Logistic regression was used to compare the parameters with the end of induction RT-PCR values. The probability of survival was estimated with the use of the product-limit method of Kaplan–Meier for overall survival (OS) and event free survival (EFS), and the significance was assessed by the log-rank test. All survival estimates are reported as ±1SE. All *p*-values were 2-sided, with values of 0.05 or less indicating statistical significance. Statistical analysis used the SPSS 16.0 Software (Chicago, IL, USA). Non-linear regression curves and graphs were done with GraphPad Prism V6 software (California, USA) for calculating IC-50 values.

## Results

### ATO Does Not Have Direct Cytotoxic Effect on NK Cells

In order to evaluate the cytotoxic effect of ATO on the malignant cell lines *in vitro*, MTT assay was done. The mean IC50 values of ATO was assessed at micromolar (μM) concentrations for all the cell lines used in this study (Table S1 in Supplementary Material). We observed that there was a wide variation in the IC50 values between different myeloid and lymphoid cell lines.

To evaluate the cytotoxic effect of ATO on NK cells at the concentrations, we used for the downstream experiments, MTT assay and a cell viability assay were done. The IC50 of NK92MI cell line was 3.84 ± 0.3μM (*n* = 4), and the 7-AAD positive cells were 1.48% when treated with 1 µM ATO for 24 h (Figure S1 in Supplementary Material). We have also checked the proliferation of NK cell line treated with 1 µM ATO for 24 h, 48 h, and 72 h. We have observed that the ATO concentration and duration of exposure used in our experiment was not cytotoxic to NK cells and did not alter the rate of proliferation (Figure S2 in Supplementary Material).

### Differential Cytolytic Activity of NK Cells Toward Leukemic Cell Lines

Next, we evaluated the cytolytic activity of NK92MI toward different malignant myeloid and lymphoid cell lines. Toward this, we have performed CFSE 7AAD cytotoxicity assay. At the highest effector (NK cells), target (leukemic cell line) ratio of 10:1, significant cytolytic activity was noted against K562 cell line (65.15 ± 6.2%, *n* = 3). The mean cytolytic activity against NB4 was 21.5 ± 3.7% (*n* = 3), and HL-60 was 12.97 ± 1.7% (*n* = 3). The mean cytolytic activity of NK cells toward all the cell lines tested was summarized in Table 1. We observed that leukemic cell lines have a differential susceptibility to the cytolytic activity of NK cell line.

**Table 1:** The percentage cytolytic activity of NK92MI cell line toward different leukemic cell lines at the E:T ratio of 10:1 assessed by CFSE/7AAD cytotoxicity assay.

Cell lines	% cytolytic activity (mean ± SD) (*n* = 3)
NB4	21.5 ± 3.7
NB4-EVAsR1	4.4 ± 0.65
UFI	16.32 ± 5.9
K562	65.15 ± 6.2
U937	10.9 ± 1.03
HL60	12.97 ± 1.7
SUP-B15	6.65 ± 2.7
Jurkat E6.1	16.7 ± 7.8

*The data are represented as mean ± SD (*n* = 3)*.

### ATO Enhances the Cytolytic Activity of NK Cells

**Figure 2:**
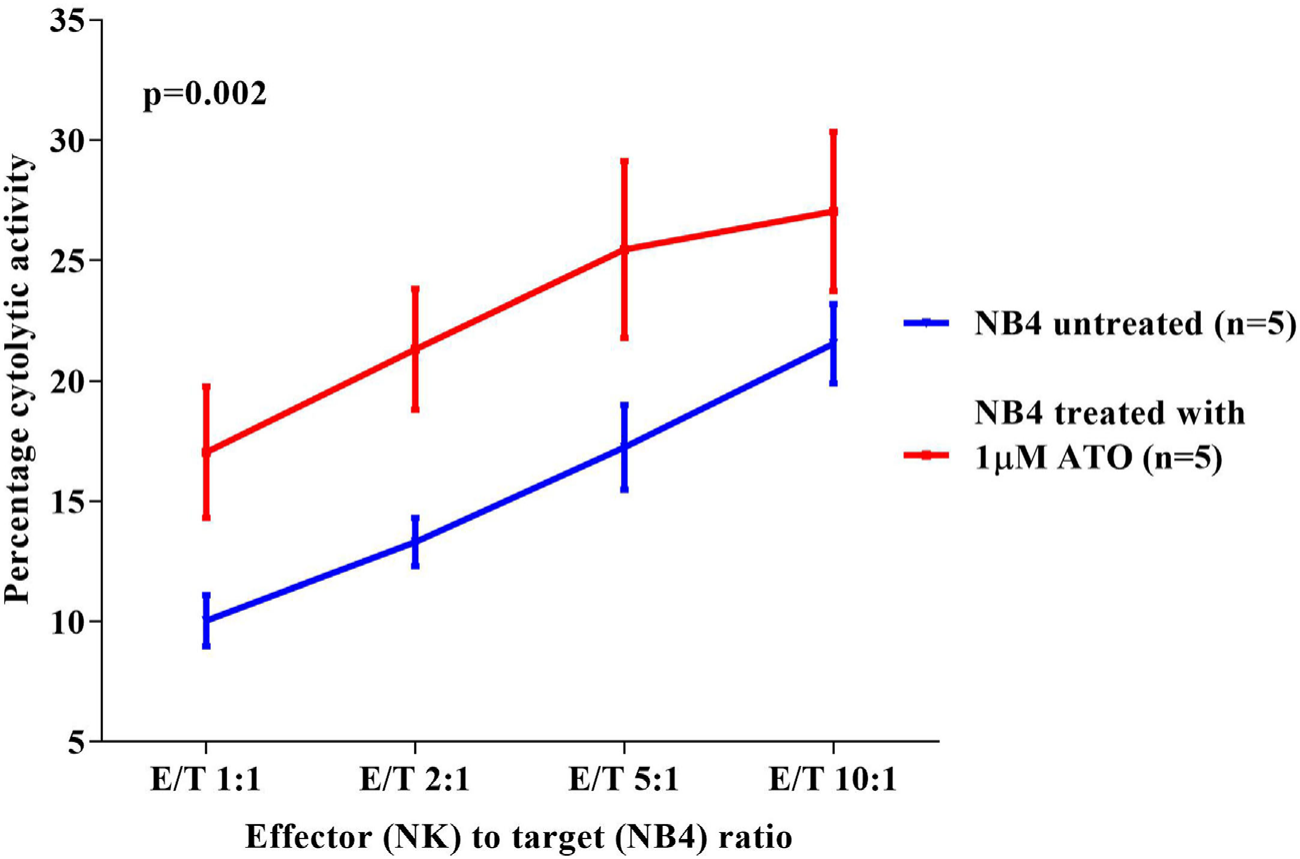
Comparison of percentage cytolytic activity of NK92MI cell line toward NB4 untreated and treated with arsenic trioxide (ATO) (1 µM for 12 h). The CFSE labeled NB4 cells (1 × 10^5^ cells untreated or treated with 1 µM ATO for 12 h) were co-cultured with effector cells (NK cell line) in different E:T ratios 1:1, 2:1, 5:1, 10:1 in a 24-well plate with 500 µl 10% RPMI media. At the end of incubation at 37°C for 5 h, the cells were washed, and 2.5 µl of 7AAD was added to the samples and acquired in FACS Calibur. The percentage cytolytic activity was calculated, and the values were plotted as mean with SEM (*n* = 5). *p*-value less than 0.05 was considered significant.

Since a significant NK cytolytic activity was observed against NB4, we next checked the effect of ATO on the NK cytotoxic activity toward these cell lines. We observed that NB4 cells when treated overnight with 1 µM ATO (>99% viability retained after this exposure) significantly increased the cytotoxic effect of NK92MI at all E:T ratios evaluated (*p* = 0.002) with a mean percentage cytolytic activity of 27.04 ± 7.3% (*n* = 5) at the highest E:T ratio of 10:1 (Figure 2). A similar cytolytic pattern was seen with other cell lines though it was not as prominent or significant as seen with NB4 cells (data not shown).

**Figure 3:**
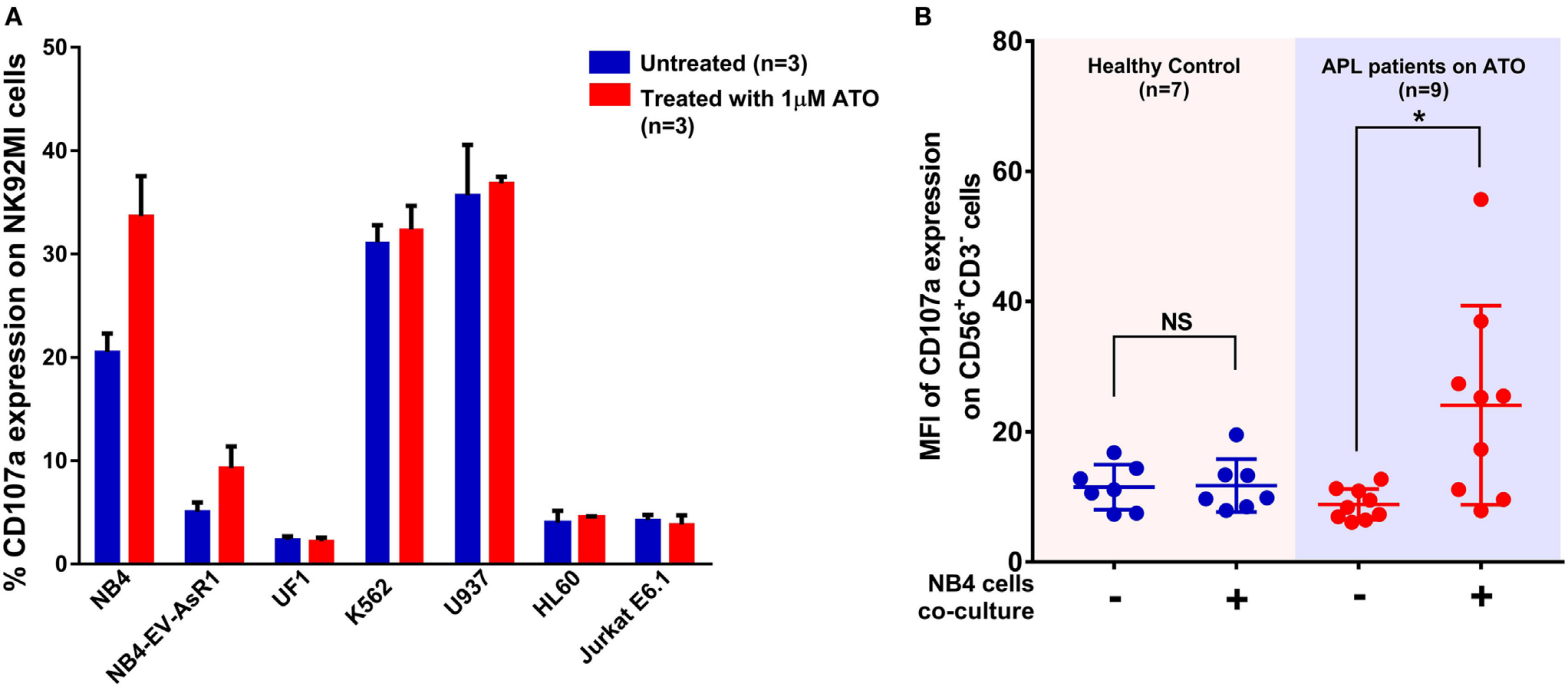
Arsenic trioxide (ATO) enhances the cytolytic activity of NK cells. **(A)** Bar graphs showing the percentage expression of CD107a (a marker for degranulation of NK) when NK cell lines were cocultured with leukemic cell lines at the ratio of 2:1 for 5 h. The blue bars represent the experiment with untreated leukemic cell lines and the red bars represent the experiment after exposure of leukemic cell lines to 1 μM ATO for 12 h (*n* = 3). **(B)** Graph shows the mean fluorescence intensity (MFI) of CD107a expression gated on CD56^+^CD3^−^ cells obtained from PBMNCs of healthy controls (no ATO exposure; *n* = 7) and acute promyelocytic leukemia (APL) patients (under ATO treatment; *n* = 9) with and without co-culture of NB4 cells at the ratio of 2:1 for 5 h.

As CD107a is a functional marker for NK cell activity, we performed CD107a degranulation assay to evaluate the effect of ATO on NK cell activity toward these cell lines. At an E:T ratio of 2:1 (*n* = 3), the mean percentage of CD107a with NB4 cell line without ATO treatment was 20.4 ± 1.8% at the end of 5 h. Whereas, when treated with 1 µM of ATO for 12 h there was a significant increase in the percentage of CD107a (33.6 ± 3.8%, *n* = 3) (Figure 3A). The mean percentages of CD107a of K562, U937, HL60, and Jurkat were 31.3 ± 1.6, 35.6 ± 4.8, 4 ± 1.13, and 4.2 ± 0.5%, respectively and of the resistant cell lines NB4-EVAsR1 and UF1 were 5.08 ± 0.8 and 2.33 ± 0.33%, respectively. Exposure of these cell lines with 2 µM ATO did not significantly increase the CD107a expression (data not shown). We were also able to demonstrate this effect of ATO on NK cells (CD56^+^CD3^−^) *in vivo* by showing an increased expression of CD107a in APL patients (undergoing ATO treatment) when co-cultured with NB4 cells compared to healthy controls (no ATO exposure) (Figure 3B; Figure S3 in Supplementary Material).

### ATO Alters NK Cell Receptor and Ligand Profile

**Figure 4:**
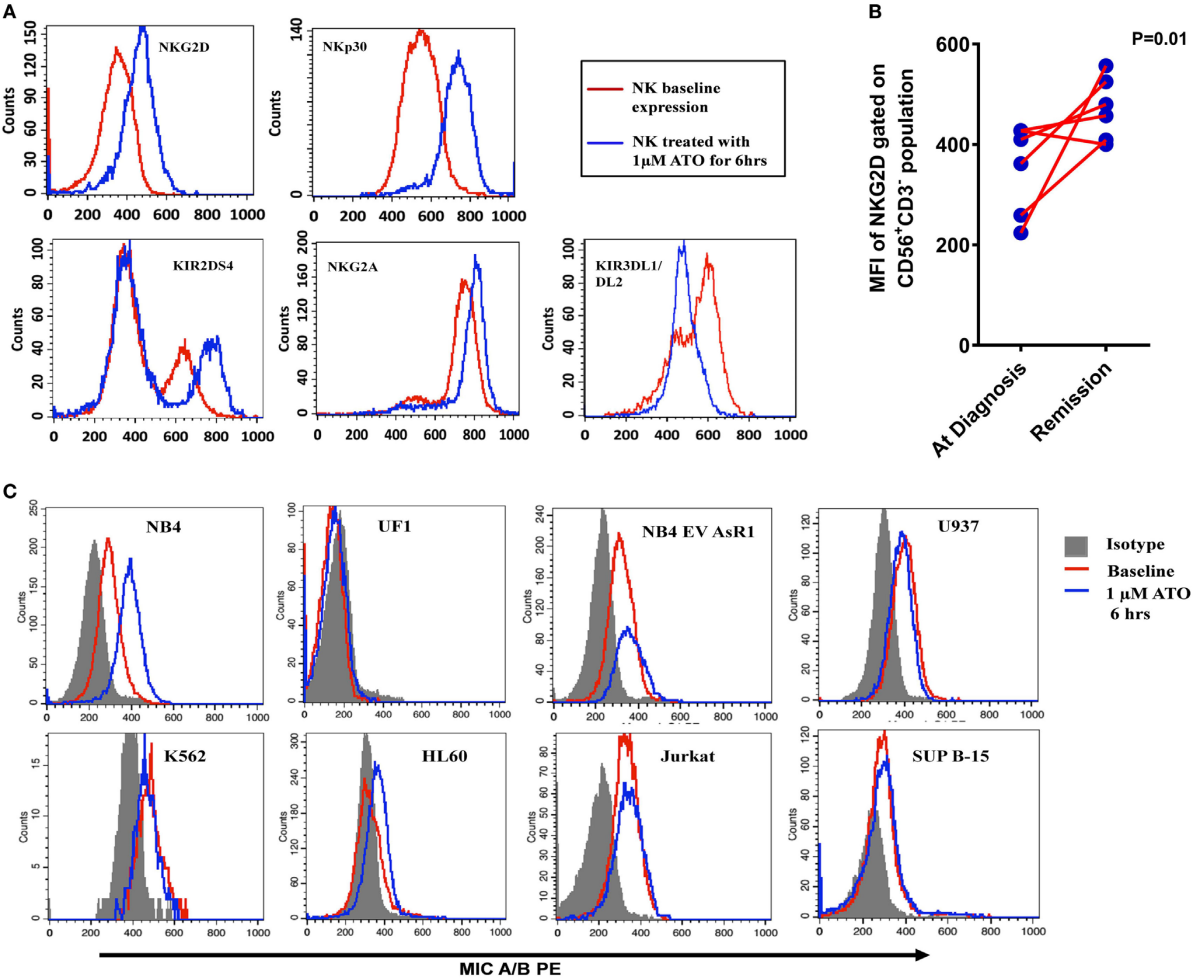
Alteration of NK receptor and ligand profile by arsenic trioxide (ATO). **(A)** Representative histogram plots of NK activating receptors NKG2D, NKp30, and KIR2DS4 and NK inhibitory receptor NKG2A of NK cell line (NK92MI) treated with 1 µM ATO for 6 h (*n* = 5). The red lines represent the baseline expression and the blue lines which show a shift toward right indicated the increased expression with 1 µM ATO treatment for 6 h. **(B)** Mean fluorescence intensity (MFI) of NKG2D receptor expression (gated on CD56^+^CD3^−^ population) in PBMNCs of acute promyelocytic leukemia (APL) patients at diagnosis and on remission (*n* = 6). **(C)** Representative histogram plots of MICA/B expression in myeloid and lymphoid cell lines treated with 1 µM ATO for 6 h (*n* = 3). The shaded region represents the isotypic controls and the red line represents the baseline expression of ligands. The blue lines which show a shift toward the right in NB4 indicating increased expression of MICA/B after ATO treatment which was not observed in other cell lines.

In order to study the mechanism of increased cytolytic activity of NK cells in the presence of ATO, we checked the NK receptors and ligand profiles upon exposure to ATO by flow cytometry. We identified that NK92MI cell line upon 6 h of exposure 1 µM ATO resulted in increased expression of activating receptors NKG2D, NKP30, and KIR2DS4 and inhibitory receptor NKG2A and decrease in inhibitory receptors KIR3DL1/DL2 (Figure 4A). The cells also retained 99% viability at the end of 24 h by 7AAD assay. There were no changes in the expression of NKP46, KIR2DL1, KIR2DL2, and DNAM1 receptors (data not shown). Increased concentration of ATO (2 µM) or increased exposure time (24 h) did not further increase the expression of these markers. Similar increased expression of NKG2D receptor was observed in NK cells from APL patients on remission when compared to their matched diagnosis samples following treatment with ATO. The median of NKG2D receptor’s mean fluorescence intensity (MFI) was 386 (224–428) at diagnosis and on remission it increased to 468.6 (400–557) (*n* = 6, *p* = 0.01) (Figure 4B; Table S2 in Supplementary Material).

We also evaluated the effect of exposure of leukemic cell lines to 1 µM ATO for 6 h on NK ligand expression by flow cytometry. There is a significant increase in activating ligand MICA/B in NB4 cell line (*n* = 3; *p* = 0.016) and a marginal increase in HL60 when compared with other malignant cell lines (Figure 4C) on exposure to ATO. Similarly, there was a significant increase in the expression of CD112/Nectin-2 (DNAM-1 ligand) and HLA Class I in NB4 cell line on treatment with ATO (Figure S4 in Supplementary Material). There was no further increase in the expression of the above ligands when treated with increased concentrations or with increased exposure time.

### NK Cellular Therapy With ATO Prolong the Survival in APL Mouse Model

**Figure 5:**
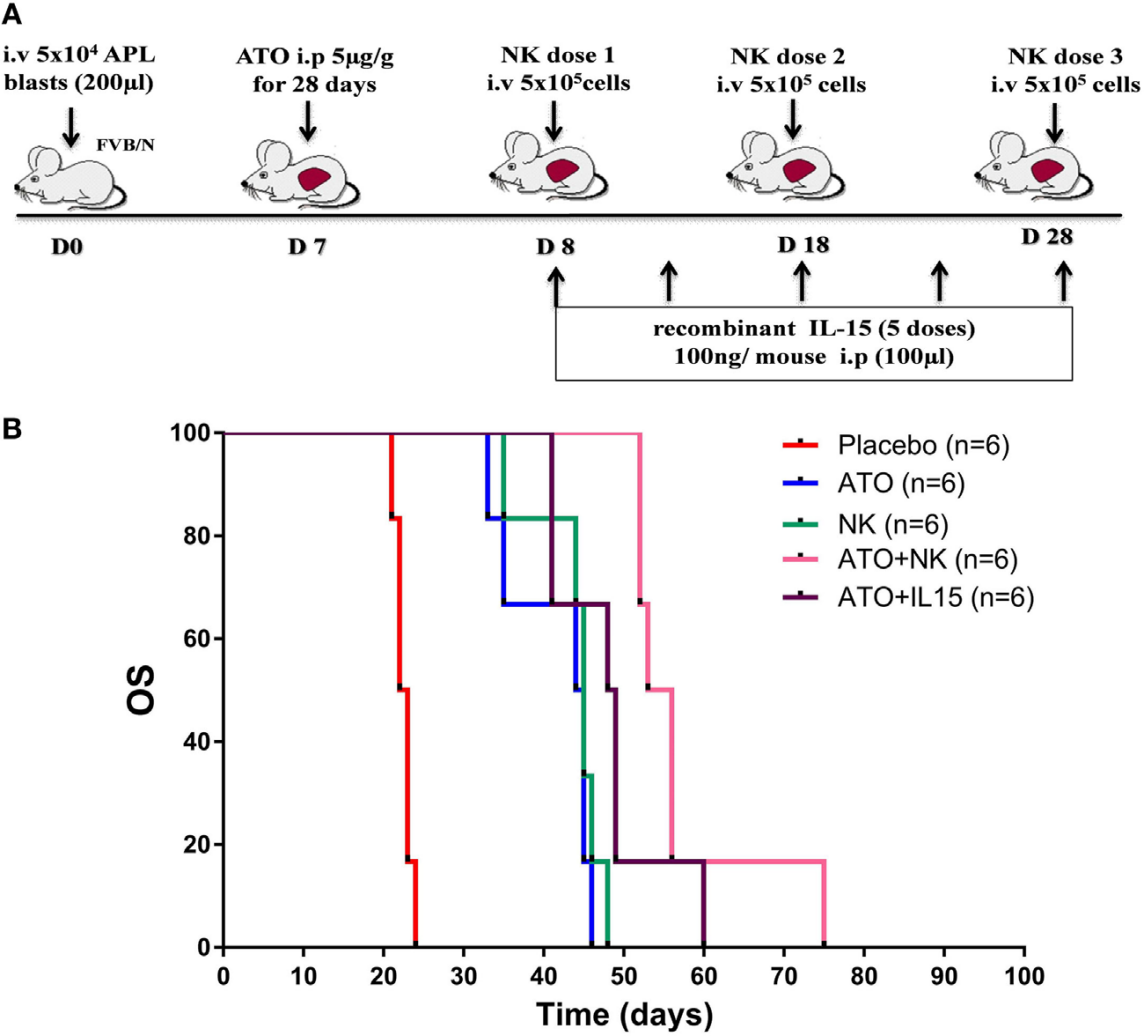
NK cell therapy improved the overall survival in acute promyelocytic leukemia (APL) mice. **(A)** Schematic representation of NK cell therapy in APL mouse model. The time points and doses of administration of arsenic trioxide (ATO), NK cells, and IL-15 were mentioned. Mouse APL cells (5 × 10^4^ cells/mouse) were injected intravenously *via* tail vein into wild-type FVB/N (Day 0). ATO (5 µg/g intra-peritoneal) was given from day 7 for 28 days. NK cells were sorted from the spleen of wild-type FVB/N and given intravenously (5 × 10^5^ NK cells/mouse, 3 doses) from day 8 with 10 day intervals. Recombinant mouse IL-15 (100 ng/mouse, 5 doses) was administered intraperitoneally from day 8 with 5 day intervals. **(B)** Survival curve showing FVB/N treated with ATO, NK cells alone, ATO + NK cells, ATO + IL15, and placebo (*n* = 6/arm). Mice treated with ATO along with 3 doses of NK shows a significantly increased survival (median survival 54 days, range: 52–75 days) when compared with ATO alone (median survival 44 days, range: 33–46 days) (*p* = 0.000). *p*-value less than 0.05 was considered significant.

Since we have observed the role of ATO in inducing the functional activity of NK cells, we then assessed the ability of NK cells in extending the survival in APL transplantable model (Figure 5A). Leukemic mice treated with NK cells alone did not show any improvement in survival when compared with the group treated with ATO (Figure 5B). Whereas, when ATO was given along with 3 doses of NK cells shows a significantly increased survival with a median survival of 54 days (range: 52–75 days) when compared with ATO group with a median survival of 44 days (range: 33–46 days) (*p* = 0.0006) (Figure 5B). Addition of IL-15 along with ATO and NK cells had an added survival advantage in comparison to the group treated with ATO + NK cells even though not statistically significant (*p* = 0.328) (Figure S5 in Supplementary Material).

### Genotype Analysis of Activating and Inhibitory KIR Genes

To evaluate the expression of KIRs in APL patients who received treatment with single agent ATO based regimen, a standard KIR genotyping assay was done (*n* = 55). The median follow-up of this cohort was 46 months and 16 cases relapsed following initial therapy. All the 16 KIR genes (6 activating receptors KIR2DS1, KIR2DS2, KIR2DS3, KIR2DS4, KIR2DS5, KIR3DS1 and 8 inhibitory receptors KIR2DL1, KIR2DL2, KIR2DL3, KIR2DL4, KIR2DL5, KIR3DL1, KIR3DL2, KIR3DL3) and 2 pseudo genes (KIR2DP1, KIR3DP1) have been screened in all the patients (Table 2). Out of the 55 patients screened, 18.2% (*n* = 10) of the patients had A haplotype and 81.2% (*n* = 45) patients had B haplotype. On a univariate analysis, there was no specific association with any specific genotype or haplotype with the risk of relapse or any other clinical outcome parameter. However, there was a trend toward significance for KIR2DL2 inhibitory receptor in relapse patients (13 relapse patients out of 16 are positive for KIR2DL2) when compared to non-relapse group (*p* = 0.069) with a hazard ratio of 3.2 (95% CI: 0.91–11.26) (Table S3 in Supplementary Material).

**Table 2:** Table showing the presence or absence 16 KIR genes screened in APL patients (*n* = 55).

KIR genes	Number of APL patients (*n* = 55)	
	Positive	Negative
2DL1	55	0
2DL2	34	21
2DL3	44	11
2DL4	55	0
2DL5 A/B	40	15
2DS1	33	22
2DS2	32	23
2DS3	27	28
2DS4 Del	42	13
2DS4 Ins	19	36
2DS5	32	23
3DL1	45	10
3DL2	55	0
3DL3	55	0
3DS1	32	23
2DP1	55	0
3DP1	55	0

*The framework genes KIR2DL4, KIR3DL2, KIR3DL3 and KIR3DP1, the pseudogenes KIR3DP1 and KIR2DP1 and KIR2DL1 is present in all the patients and controls*.

*KIR, killer immunoglobulin-like receptor; APL, acute promyelocytic leukemia*.

### Delayed Recovery of NK Cells in Newly Diagnosed APL Patients Treated With Single Agent ATO

**Figure 6:**
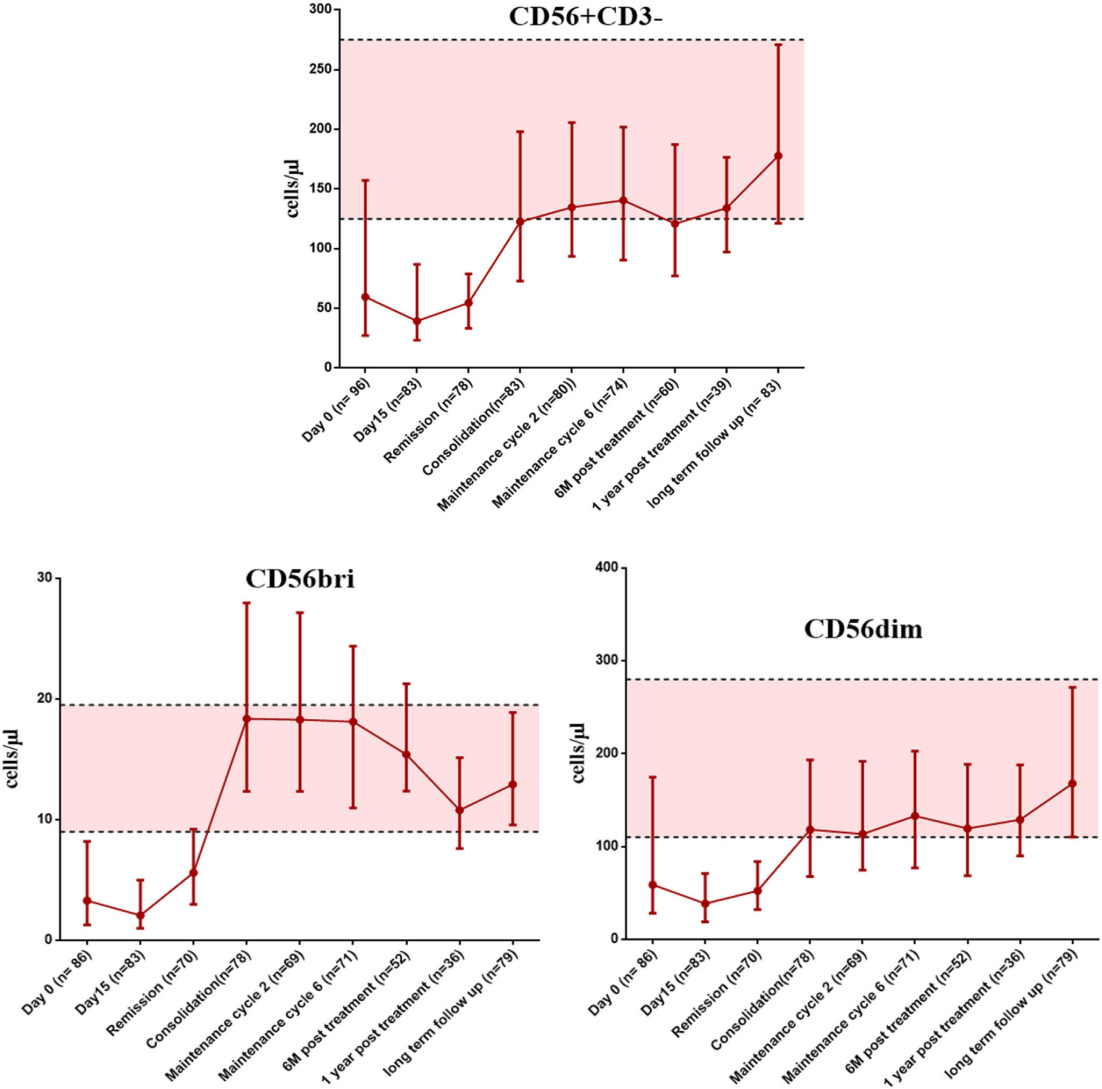
The graphs showing the recovery of NK cells and it subsets (CD56^bright^ and CD56^dim^) at appropriate time points post treatment with arsenic trioxide. The error bars indicate median with interquartile ranges. The shaded region represents the 25th to 75th percentile values of very long term follow-up samples serve as normal reference ranges. Number of samples available at each time points were mentioned in brackets. The time points of samples collected were mentioned on X axis. Absolute counts were given as cells per microliter on Y axis.

We then looked at the immune reconstitution pattern of immune subsets in APL patients treated with single agent ATO at different time points of therapy (clinical details of the patients were given in Supplementary Results S1 in Supplementary Material and Table S4 in Supplementary Material). Following treatment with ATO, there was a differential pattern of immune reconstitution in different lymphocyte subsets (Figure 6; Figure S6–8 in Supplementary Material). The time span for circulating NK cells to achieve the median normal levels was beyond 655 days (1 year post treatment) when compared with T cells and B cells [145 days (at maintenance cycle 2) and 265 days (at maintenance cycle 6) respectively]. We observed a significant delay in the reconstitution of NK cells (median absolute counts at 6 months post treatment was 120.84 cells/μl, range: 27.85–484.22 cells/μl vs the long term follow-up samples median 177.85 cells/μl, range: 61.02–635.37 cells/μl) (Table S5 in Supplementary Material). We have also looked at the reconstitution pattern of two major subsets of NK cells, CD56^bright^, and CD56^dim^. The median absolute counts of CD56^dim^ subset were lower throughout the course of treatment when compared with CD56^bri^ subset (Figure 6). The CD56^+^CD3^+^ NKT subset lies almost in the normal limits till the end of maintenance.

Looking at the recovery pattern of other subsets, the earliest recovery to the normal range was seen in the CD3^+^CD8^+^ T cytotoxic cells, and the CD4/CD8 ratio remains inverted until the start of consolidation therapy. The absolute counts of the immune subsets at different time points of treatment were given in Tables S5 and S6 in Supplementary Material.

### Impact of NK Cell Subset Reconstitution on Clinical Outcomes of Patients Treated With ATO

**Figure 7:**
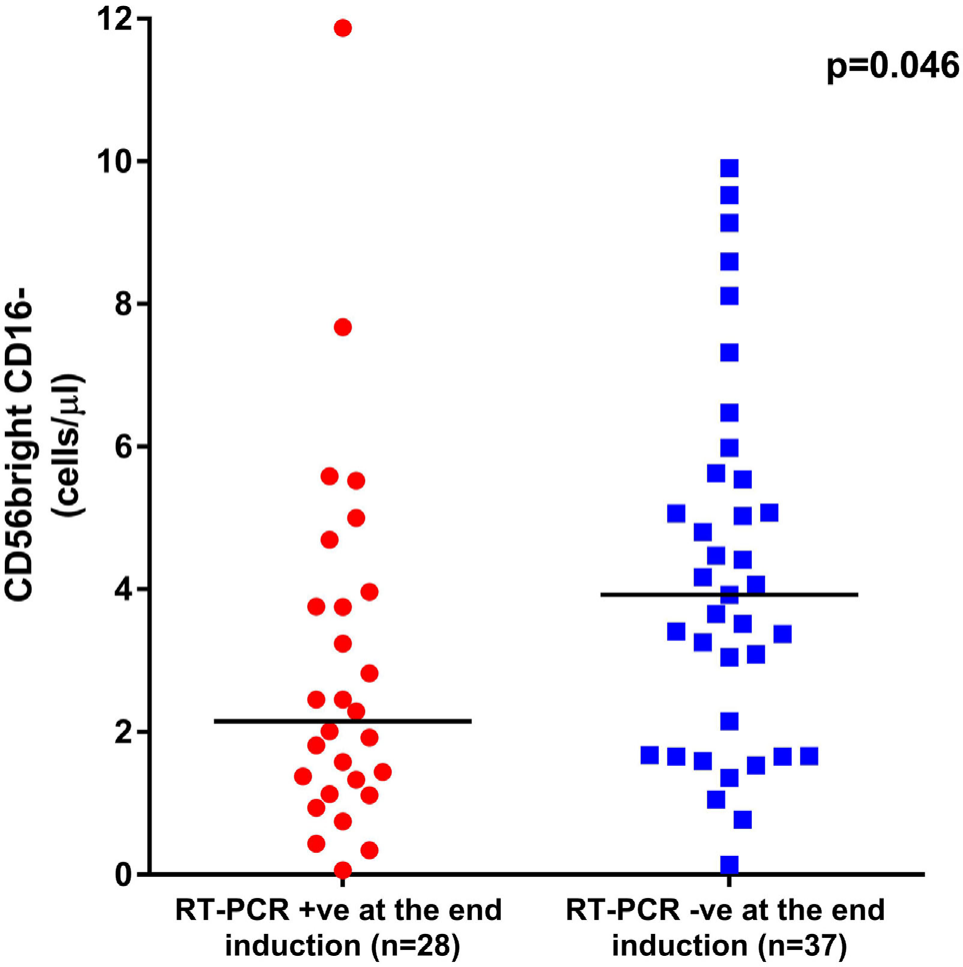
Graphs showing the impact of CD56^bright^ CD16^−^ subset recovery on clinical outcome. The median absolute counts of CD56^bright^ CD16^−^ immature subset was significantly lower in those patients who were RT-PCR positive at the end of induction (*n* = 28) with a median of 2.15 cells/μl (range: 0.06–11.87 cells/μl) when compared with those who were RT-PCR negative (*n* = 37) with a median of 3.92 cells/μl (range: 0.14–9.9 cells/μl, *p* = 0.046).

Since we observed a delayed NK cell recovery on treatment with ATO, we looked at the impact of this NK maturation pattern on clinical outcomes of APL patients. At the end of induction, the absolute counts of CD56^bright^ CD16^−^ immature subset were significantly lower in those patients who were RT-PCR positive (*n* = 28) with a median of 2.15 (range: 0.06–11.87) cells/μl compared with those who were RT-PCR negative (*n* = 37) with a median of 3.92 (range: 0.14–9.9) cells/μl (*p* = 0.046) (Figure 7). However, the NK reconstitution pattern did not correlate with EFS or OS. No other subsets evaluated at the end of induction were significant (data not shown).

### Assessment of NK Differentiation From CD34 Cells With and Without ATO Treatment

Even though ATO was shown to have modulating the NK cell cytolytic activity, there was a defect in the NK cell recovery. Hence, we hypothesized that ATO may have an impact on normal hematopoietic stem cell (HSC) ability to differentiate and mature into NK cells accounting for this delay in reconstitution. Hence, we assessed the NK cell differentiation from CD34 cells *in vitro* with and without exposure to 0.5 µM ATO by flow cytometry. We observed that 5.1 ± 0.3% cells were positive for CD56^+^CD3^−^ NK on day 8 and it increased to 7.7 ± 0.4% on day 14 in the culture without ATO. Whereas, CD34 sorted cells seeded in media with ATO had only 2.3 ± 0.4% CD56^+^CD3^−^ cells on day 8 (*n* = 3, *p* = 0.017) and 5.2 ± 0.4% on day 14 (*n* = 3, *p* = 0.038) showing a delay in NK differentiation *in vitro*. There was a slight increase in the myeloid compartment on day 14 in culture whereas CD3 and CD19 remained the same (Figure S9 in Supplementary Material).

### Role of Transcription Factors Involved in NK Cell Differentiation

**Figure 8:**
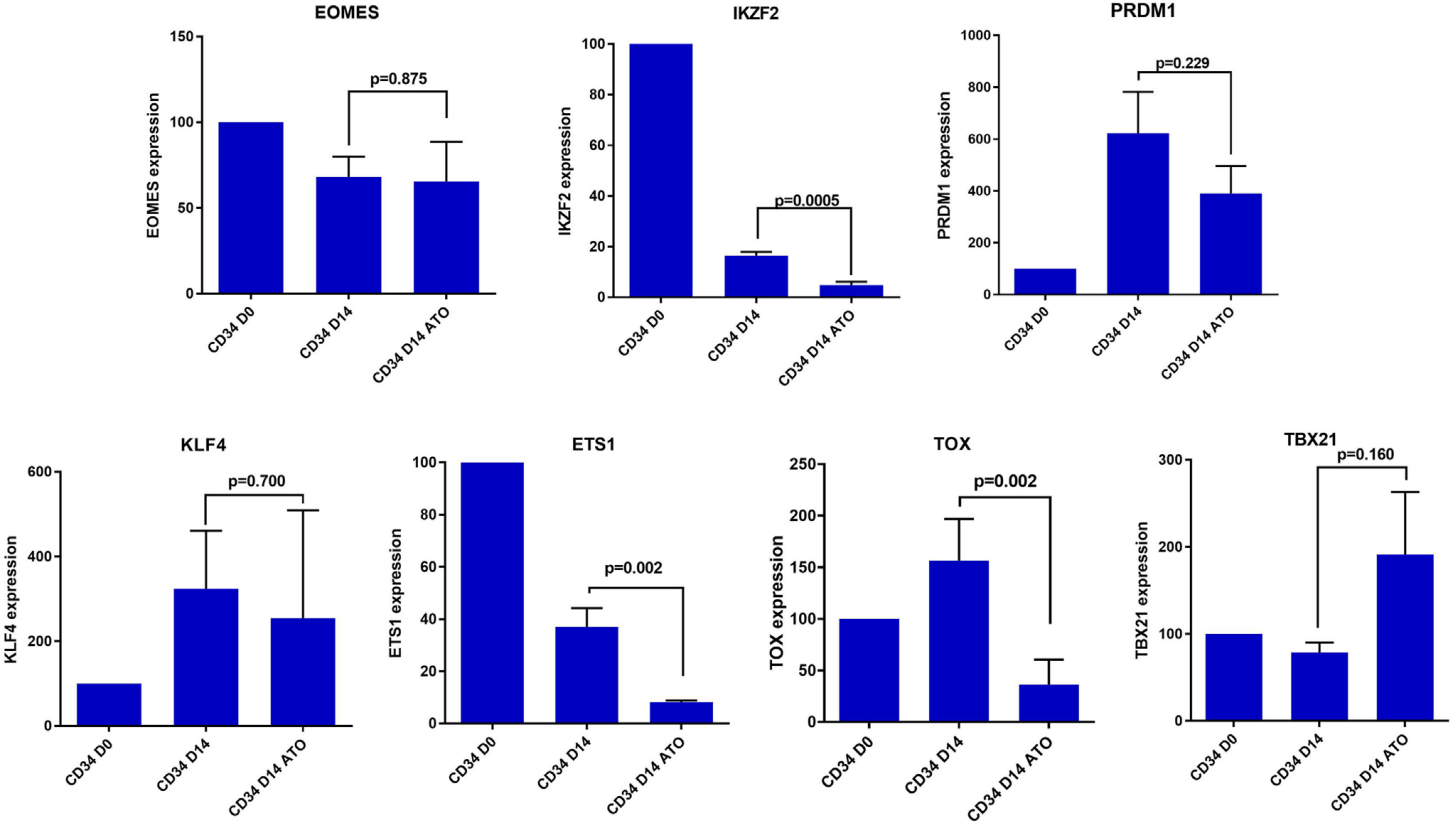
Expression of differentially expressed transcription factors involved in NK cell differentiation and maturation by RQ-PCR. The expression of the genes were determined by 2^ΔΔCT^ method. The expression of these genes were normalized to GAPDH and the relative expression of genes on CD34 on day 14 (±ATO) was calculated with CD34 day 0 sample (*n* = 3). *p*-value less than 0.05 was considered significant.

In view of this quantitative defect in NK cells, we next evaluated the role of transcription factors contributing to NK differentiation and maturation. Even though the precise hierarchy of the transcription factors that control NK cell maturation is not known, we evaluated the expression of some of the major transcription factors like *EOMES, IKZF2, PRDM1, ETS1, TOX, KLF4*, and *TBX21* which are involved in the differentiation of CD34 cells to mature activated NK cells (34, 35). We observed a significant decrease in the expression of TFs *IKZF2, ETS 1*, and *TOX* in day 14 CD34 cells treated with ATO when compared to untreated (*p* = 0.0005, *p* = 0.002, and *p* = 0.002 respectively), which are shown to be involved in the transition from pre NK cells to immature NK (Figure 8). *TBX21* which is also involved in maturation of NK cells was not downregulated on treatment with ATO.

## Discussion

There is accumulating evidence of anti-cancer chemotherapy additionally being involved in augmentation of host immune reactivity (36). Earlier publications from our center showed the efficacy of single agent ATO in terms of durable remissions and minimal toxicity for the treatment of newly diagnosed APL patients (31). Studies were done looking at the ability of malignant promyelocytes to concentrate ATO intracellularly (37) and the role of ABC transporters involved in ATO efflux (38). Also, our group has looked at the innate environment mediated drug resistance to ATO in APL leukemic cells (24). Some studies have reported that ATO binds directly to cysteine residues and has shown direct interaction between ATO and the PML protein (39). Other than the known cellular mechanisms of ATO, its role in modulating immune system is less explored.

In this study, we tried to understand the effect of ATO, if any, in modulating the immune responses. Our initial work related to immune reconstitution following treatment of APL with single agent ATO illustrated an unusual pattern of delay in NK cell recovery post completion of treatment. This is contrast with our earlier reported data on NK cell recovery post-allogeneic stem cell transplantation where we noted that NK cells were the first subset to recover (28). Similarly, in many immune reconstitution studies post chemotherapy or post-allogeneic stem cell transplantation, it is usually the cells of the innate system that are the first to reconstitute (23, 30). In this study, the reconstitution pattern of NK cells was distinct and much delayed after exposure to ATO. To the best of our knowledge, this is the first time this observation has been made. This delay in reconstitution could not be explained by a direct cytolytic effect of ATO on NK cells as demonstrated by us. Additionally, as reported by us previously, the patients were treated with single agent ATO, and hence, this delay in reconstitution could not be attributed to any other chemotherapeutic agent. This delay in reconstitution persisted even after completing therapy and patient being in molecular remission with an otherwise normal bone marrow study. Immune reconstitution of other cellular subsets was as expected and consistent with previously reported studies. Further, our analysis has shown that among the CD56 subsets, the median absolute counts of CD56^dim^ populations were lower and recovered slowly than the CD56^bright^ population in our cohort. Since CD56^bright^ are the immediate precursors of the CD56 dim subset, we think there is a delay in the differentiation of CD56^bright^ to CD56^dim^ population probably mediated by the exposure with ATO. Our data suggest the effect we observed is more likely to be an effect of ATO on normal differentiation and maturation of NK cells from HSC *in vivo*. Consistent with this hypothesis, we noted that ATO had no direct cytotoxic effect on NK cells *in vitro* nor did it alter the rate of proliferation of NK cells. We further evaluated the effect of ATO on normal CD34^+^ HSC specifically looking at its effect of expression of transcription factors involved with NK cell development and differentiation (40, 41). We noted a significant decrease in the expression of *TOX* and *ETS-1* on day14 in CD34 cells treated with ATO when compared to untreated. These factors are critical for NK cell maturation and differentiation.

In contrast to the effect of ATO in delaying NK cell reconstitution post treatment, we also noted that ATO enhances NK cell-mediated cytolytic activity against leukemia cells. We confirmed this increased cytolytic activity of NK cells by performing CD107a degranulation assay (42). We also noted that this enhance NK cell cytolytic activity was predominantly seen with myeloid cell lines and very little to no effect with lymphoid cell lines.

The diversity of receptors and ligands of NK cells determine its ability to eliminate a defective target (43). We hypothesized that the cytolytic activity is mediated by the presence or absence of activating and inhibitory NK receptors and ligands which could potentially be modulated by ATO. Studies have shown reduction of MICA/B surface expression that may impair NKG2D-mediated immune surveillance of leukemia (44). Our experimental data suggest that ATO alters the NK cell receptor and malignant cell ligand profile in a direction that enhances NK cell mediated cytolytic activity. Similar observation was seen in one other study showing upregulation of NKG2D ligands by ATO in K562, NB4, and MCF7 breast cancer cell lines and increased susceptibility to NK-mediated cytotoxicity (25). Our data suggest that there is also additional modulation of the expression of the NK cell receptors by ATO and this effect could potentially be exploited to enhance NK cell anti-tumor effects. Additional experiments to further validate these observations that could potentially be done and were not done as part of this manuscript, could include blocking/activating of receptors and ligands using either small molecules, blocking antibodies, or activating agents.

Translating the observation from this study, we studied the effect of NK therapy in a mouse model of APL treated with ATO, previously established in our laboratory (10). We hypothesized that infusing mouse NK cells could increase the anti-leukemic activity and hence prolong the survival of mice treated with ATO. We observed that NK cells when infused along with ATO extended the survival in APL mice. NK cells pre-activated with IL-12, IL-15, and IL-18 were shown to induce functional responses against primary AML blasts (45). Our data suggest that addition of IL-15 along with NK cells had an added advantage on survival even though not statistically significant.

We have demonstrated for the first time the role of ATO in modulating the NK cell anti-tumor activity. Low dose ATO exposure to enhance NK cell activity could potentially be exploited in strategies that use NK cell *in vitro* expansion and optimization for treatment of cancer. Administration of ATO to patients prior to NK cell infusion could also be explored as a strategy to enhance NK cell activity especially, from this body of work, in AML.

## Ethics Statement

All procedures performed in studies involving human participants were in accordance with the ethical standards of the institutional research committee and with the 1964 Helsinki declaration and its later amendments or comparable ethical standards.

## Author Contributions

AAA: performed research, designed study, performed flow cytometry, molecular tests, mouse experiments, analyzed data, and wrote paper. SG: performed research, performed molecular tests, flow cytometry, mouse experiments, and analyzed data. HP: performed research, performed flow cytometry, molecular tests, and analyzed data. NB, SD, and NJ: performed research and analyzed data. KL: performed statistical analysis. UK, PN, AK, AD, AA, AS, and BG: performed research, clinical data accrual, and analyzed data. RP and CC: performed research, mouse experiment design, and analyzed data. PB: performed research, performed molecular tests, and analyzed data. VM: performed research, designed study, clinical data accrual, analyzed data, and wrote paper.

## Conflict of Interest Statement

The authors declare that the research was conducted in the absence of any commercial or financial relationships that could be construed as a potential conflict of interest.
