# Supplementary material for: Arsenic Trioxide Enhances the NK Cell Cytotoxicity Against Acute Promyelocytic Leukemia While Simultaneously Inhibiting Its Bio-Genesis
Source: Front Immunol. 2018 Jun 14;9:1357. doi: 10.3389/fimmu.2018.01357 (PMC6010577; doi:10.3389/fimmu.2018.01357)
Supplement: Supplementary file 1 [file Data_Sheet_1.docx]

**Arsenic trioxide enhances the NK cell cytotoxicity against acute promyelocytic leukemia while simultaneously inhibiting its bio-genesis**

Ansu Abu Alex^1^, Saravanan Ganesan^1^, Hamenth Kumar Palani^1^, Nithya Balasundaram^1^,

Sachin David^1^, Kavitha M Lakshmi^1^, Uday P Kulkarni^1^, Nisham P N^1^, Anu Korula^1^, Anup J Devasia^1^, Nancy Beryl Janet^1^, Aby Abraham^1^,  Alok Srivastava^1^, Biju George^1^, Rose Ann Padua^2,3^, Christine Chomienne^2,3^, Poonkuzhali Balasubramanian^1^ and Vikram Mathews^1^*

^1^ Department of Hematology, Christian Medical College, Vellore, India

^2^ UMR-S1131, Hôpital Saint Louis, Paris, France.

^3^ Institut Universitaire d' Hématologie, Universite Paris Diderot, Paris, France.

**Supplementary file**

* Correspondence:  **Vikram Mathews**

Department of Hematology

Christian Medical College

Vellore 632004

India.

Telephone No: 91-416-2282891

Fax No: 91-416-2226449

E-mail: [vikram@cmcvellore.ac.in](mailto:vikram@cmcvellore.ac.in)

**Supplementary materials and methods**

**S1. Generation of ATO resistant NB4 cell line (NB4-EVAsR1)**

An ATO resistant NB4 cell line was generated in our laboratory ([1](#_ENREF_1)). Briefly NB4 cells were exposed to serial increasing concentrations of ATO over a period of 1 year till they were resistant to 1μM ATO. Clonal populations were further isolated by limiting dilutions and methylcellulose plating to generate the ATO resistant cell line NB4-EVAsR1. This resistant cell line was characterized in detail and the IC50 of this cell line was 3.13±0.25 µM (0.94±0.04 µM for NB4 naïve cell line). The immunophenotype of NB4-EVAsR1 was also distinct from the naïve cell line. We found that this cell line has A216V mutation which was known to cause ATO resistance.

**S2. Treatment regimen of newly diagnosed APL with single agent ATO.**

The clinical trial using ATO as a single agent was initiated in January 1998 at our institution and ATO was prepared in the pharmacy department of this institution. From December 2003, commercially available ATO was utilized (INTAS Pharmaceuticals Ltd, Matoda, Gujarat, India). 10 mg per 10ml ATO vial was diluted in 500 ml dextrose saline and administered for adults and dilution in 200 ml dextrose saline for pediatric population and was infused intravenously over 2 to 3 hours, once a day, without electrocardiographic monitoring. The schedule of single-agent ATO used in this study is summarized in Figure 1. Single-agent ATO was administered until complete hematologic remission (CR) for a maximum of 60 days. Following a 4-week interval, ATO was administered for another four weeks as a consolidation course, for those in CR. Again after a 4-week interval, for those continuing to remain in CR, single agent ATO was administered ten days a month for six months. Supportive care and monitoring were done ([2](#_ENREF_2)).

**S3. Reagents and antibodies**

Arsenic trioxide (ATO) used in this study was a kind gift from Intas Pharmaceuticals Ltd, Ahmedabad, India. For NK studies, the antibodies to NK cell receptors used were NKp30, NKp44, NKp46, NKG2D and DNAM 1, KIR2DL1, KIR2DL2, KIR2DS4, KIR3DL1/DL2 and NKG2A and ligands were CD112 (Nectin 2-ligand for DNAM-1), MICA/B (ligand for activating NK cell receptor NKG2D) and HLA Class 1. For immune reconstitution studies, following subsets were analyzed: T cells CD3^+^ , Helper T cells CD4^+^, Cytotoxic T cells CD8^+^, Naïve CD4^+^CD45RA^+^, Memory CD4^+^CD45RO^+^, Naïve CD8^+^CD45RA^+^, Memory CD8^+^CD45RO^+^, B cells CD19^+^, NK CD3^-^CD56^+^CD16^+^ and its subsets, activated Helper T cells CD4^+^CD25^+^, Dendritic cells - Monocytoid (MC) - Lin^-^HLA-DR^+^CD11c^+^and Plasmacytoid (PC) - Lin^-^HLA-DR^+^CD123. All monoclonal antibodies were procured from BD Biosciences, San Jose, CA, USA which were either tagged with FITC, PE, PerCP or APC.

**S4. NK cellular therapy in APL transplantable mouse model**

For NK cellular therapy, 6-8 week old wild-type FVB/N mice was injected intravenously in the tail with 5x 10^4^ blasts (day 0) and divided into following groups. a) Placebo, b) NK cells alone, c) ATO alone, d) ATO+NK cells (n=6 in each arm). NK cells were isolated from the spleen of wild type FVB/N using EasySep® Negative Selection Mouse NK Cell Enrichment Kit (Stem cell Technologies, Vancouver, Canada). The enriched NK cells (CD49b+CD3-) were assessed for purity by flow cytometry (BC Gallios, CA, USA) after staining with fluorochrome-conjugated antibodies against CD49b and CD3 and looked for percentage enrichment when compared with the splenocytes before sorting. ATO (5μg/g intraperitoneal ) was given from day 7 for 28 days. A total of 5x 10^5^ NK cells were injected intraperitoneal for 3 doses with 10 days interval starting from day 8. To further see whether use of IL-15 would replace NK cells, we injected the APL FVB/N with 100ng of recombinant mouse IL-15 intraperitoneal starting from day 8 for a total of 5 doses with 5 days interval in combination with ATO and NK cells (leukemic FVB/N+ ATO+IL-15, n=6 and leukemic FVB/N+ ATO+NK+IL-15, n=6) .

**S5. CD34 sorting and NK differentiation**

Umbilical cord blood samples were collected from the Department of Obstetrics and Gynaecology, Christian Medical College, Vellore after informed consent. Mononuclear cells (MNCs) were isolated by density gradient centrifugation using Ficoll-Paque plus (GE Healthcare, Uppsala, Sweden). CD34 positive selection was done using Easy Sep Human CD34 positive selection Kit (Stem cell Technologies, Vancouver, Canada). The sorted cells were again subjected to a second step of sorting the next day and the positively selected CD34 cells were resuspended in NK differentiation medium (10%RPMI+10ng SCF+30ng FLT3+50ng IL-15) and kept in an incubator at 37^o^C with 5%CO_2_. The sorted CD34 cells were checked for purity by flow cytometry. The sorted CD34 cells were also exposed to 0.5μM ATO and NK differentiation was assessed on Day 8 and Day 14 by flow cytometry.

**S6. RQ-PCR for NK cell transcription factors**

RNA was isolated from the CD34 cell culture on day 0 and day 14 which were either untreated or were exposed to 0.5μM ATO. The expression levels of NK transcription factors were determined based on TaqMan® Gene Expression Assays. Hs00172872_m1, Hs00212361_m1, Hs00153357_m1, Hs00358836_m1, Hs00428293_m1, Hs01055573_m1, Hs00203436_m1 were used for detecting *EOMES, IKZF2, PRDM1, KLF4, ETS1, TOX* and *TBX21* mRNA expression respectively and was normalized to the house keeping gene *GAPDH* (Taqman assay ID Hs 02758991_g1). Gene expression was calculated by 2^ΔΔCT method where the difference in threshold cycle (ΔCT) values of the target gene and the housekeeping gene *GAPDH* for each sample was normalized to ΔCT value of the untreated sample (CD34 day0).

**Supplementary results**

**S1. Details of newly diagnosed APL patients treated with ATO**

One hundred and twelve patients diagnosed with APL in our department from March 2010 to May 2015 were prospectively enrolled in the study after getting written and informed consent. Among them, 9 patients had an early death prior to the starting of treatment (less than 7 days) and 7 patients refused treatment at our center and were discharged against medical advice (DAMA). 96 newly diagnosed patients were included in the reconstitution study. There were 47 males (48.9%) in the cohort with a median age of 32 years (range: 2-59 years). All the 96 patients received ATO as a single agent as a part of the treatment as previously reported by us ([2](#_ENREF_2)). Among the 96 patients 6 died during induction therapy and 24 relapsed (25%). All the 24 had relapsed in the bone marrow, while 12 cases had a concurrent extramedullary relapse in the CNS. Of the 24 patients who relapsed 21 received salvage chemotherapy (one patient died before treatment and 2 were discharged against medical advice). 9 patients were consolidated with autologous stem cell transplantation and the rest were treated with combination of ATRA and ATO. The Kaplan-Meier estimate of event free survival and overall survival at 3 years was 66.7± 5.2% and 91.4± 2.9% respectively.

**Supplementary figures**

**Supplementary Figure S1.** Cytotoxic effect of ATO on NK92MI cell line measured by MTT assay and by 7AAD. a) Graph representing the *in vitro* sensitivity assay of NK92MI towards increasing concentration of ATO (post 48 hours incubation) using an MTT assay. The IC50 was generated using graph pad prism software (n=4). IC50 of NK92MI cell line was 3.8±0.3μM. b) The dot plot showing 1.48% cells positive for 7AAD when NK92MI cell line treated with 1μM ATO for 24 hours*.*

**
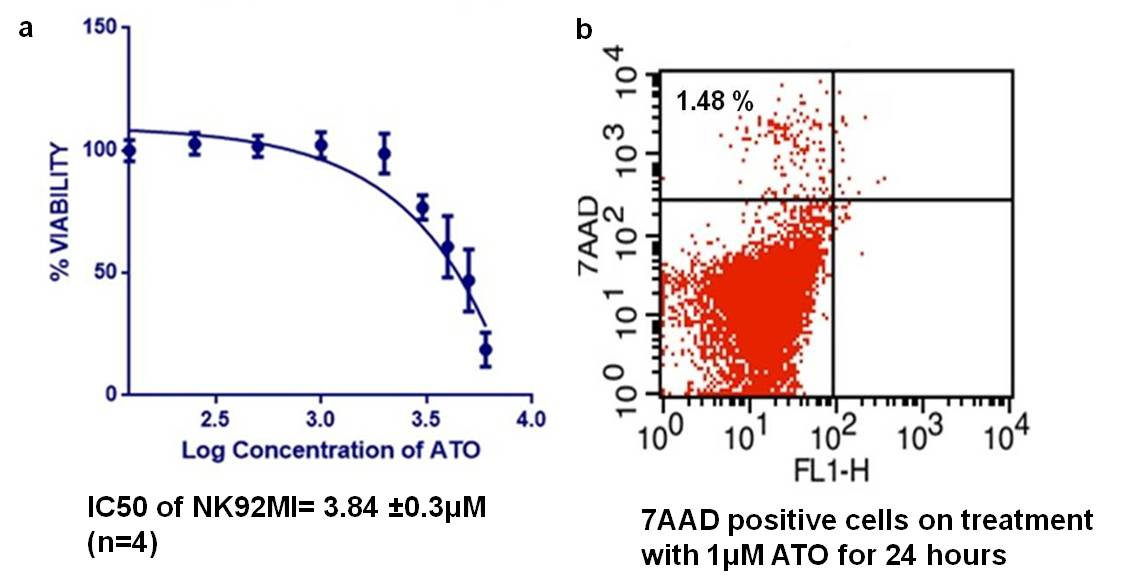
**

**Supplementary Figure S2**. Representative histogram plots showing the proliferation of NK92MIcell line at 24, 48 and 72 hours by measuring the reduction in intensity of CFSE. The shaded region represents the intensity of CFSE of NK cell line untreated and the red line indicated the intensity of CFSE of NK cell line treated with 1μM ATO.


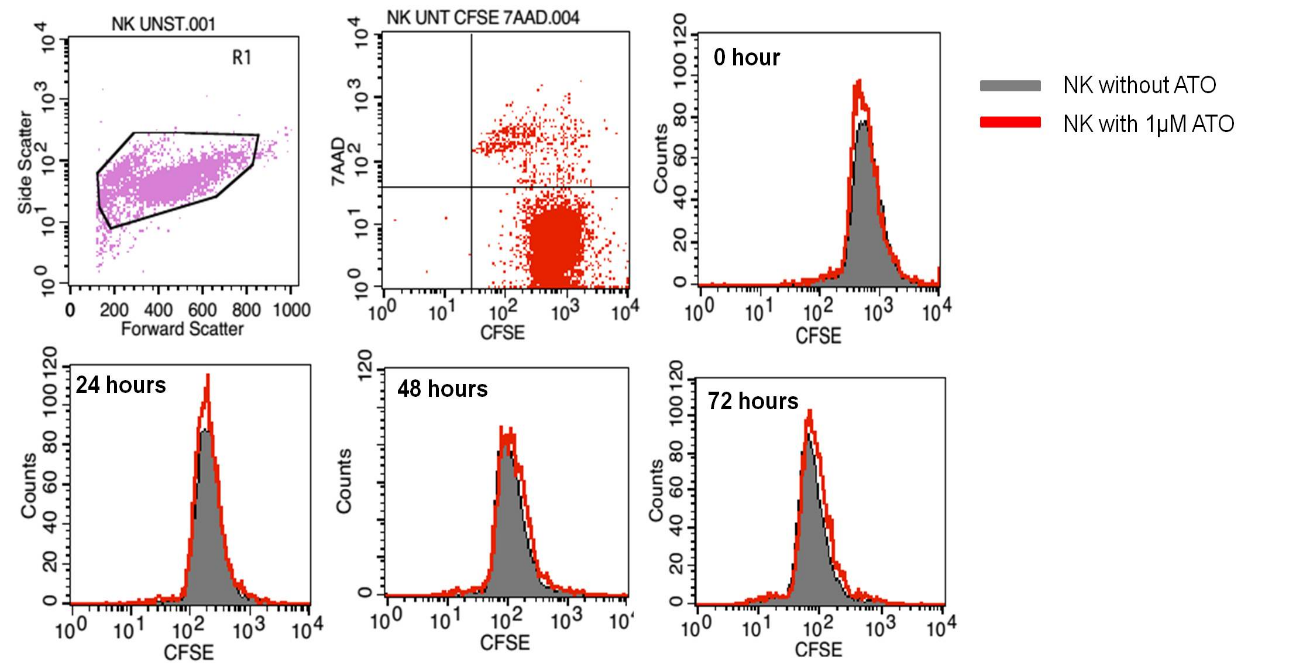


**Supplementary figure S3**. Representative histogram plots of Nectin-2, MIC A/B, and HLA Class I NK activating and inhibitory ligands on NB4 treated with 1μM ATO for 6 hours(n=3). The shaded region is the isotypic controls, and the green line represents the baseline expression of all ligands. A shift towards the right in all plots (blue lines) shows an increase in expression of ligands after ATO treatment.


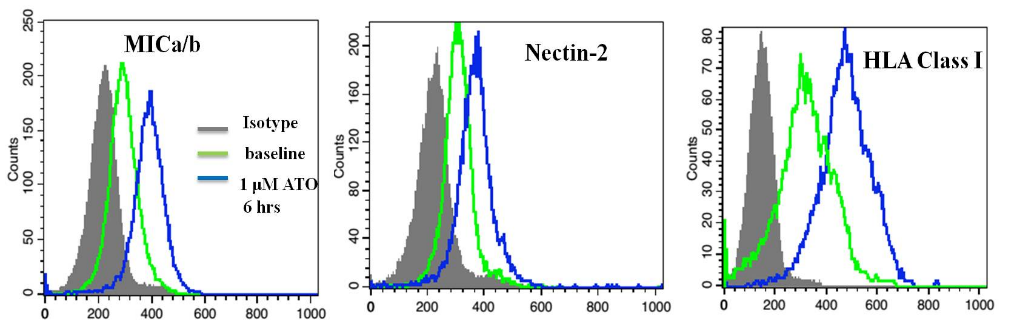


**Supplementary figure S4**. NK cell therapy improved the overall survival in APL mice. Survival curve showing FVB/N treated with ATO, ATO+NK, ATO+NK+IL15 and placebo (n=6 / arm). Mice treated with ATO along with 3 doses of NK shows a significantly increased survival (median survival 54 days, range: 52-75 days )when compared with ATO alone (median survival 44 days, range: 33-46 days ) (p=0.000). Addition of IL-15 along with NK cells had an added advantage in survival over other groups though not significant (p=0.328). p-value less than 0.05 was considered significant.


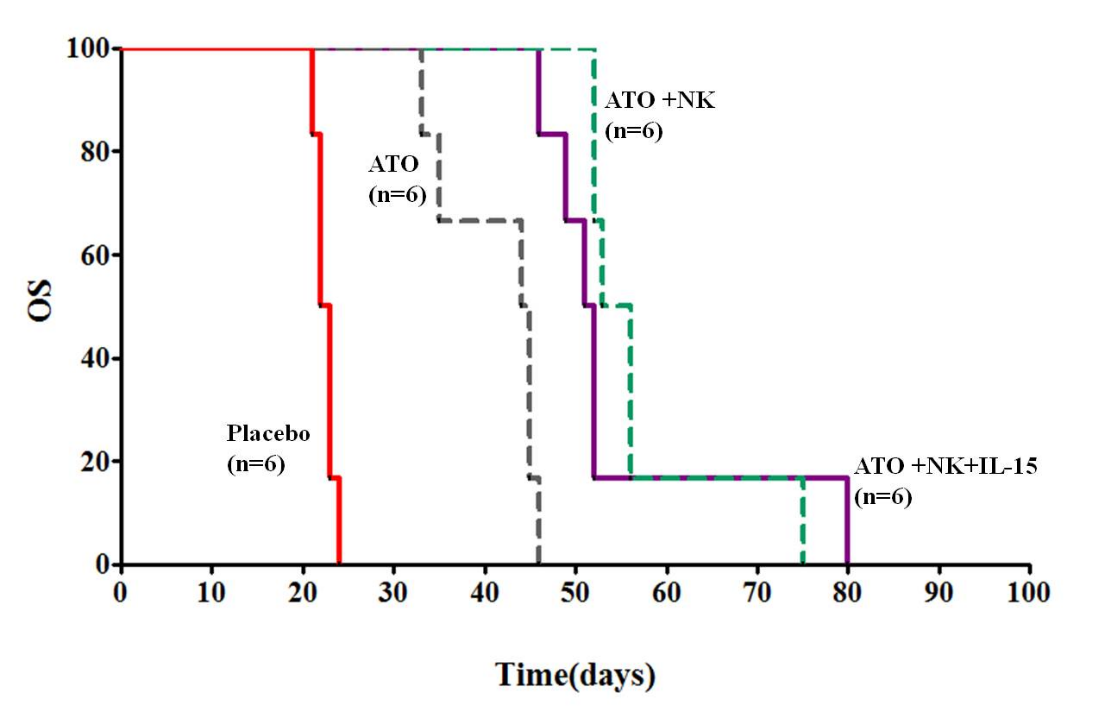


**Supplementary figure S5.** The graphs showing the recovery of T cells and B cells at appropriate time points post treatment with ATO. The error bars indicate median with interquartile ranges. The shaded region represents the 25^th^ to 75^th^ percentile values of long term follow-up samples serve as reference ranges. Number of samples available at each time points were mentioned in brackets. The time points of samples collected were mentioned on x axis. Absolute counts were given as cells per microlitre on y axis.

*
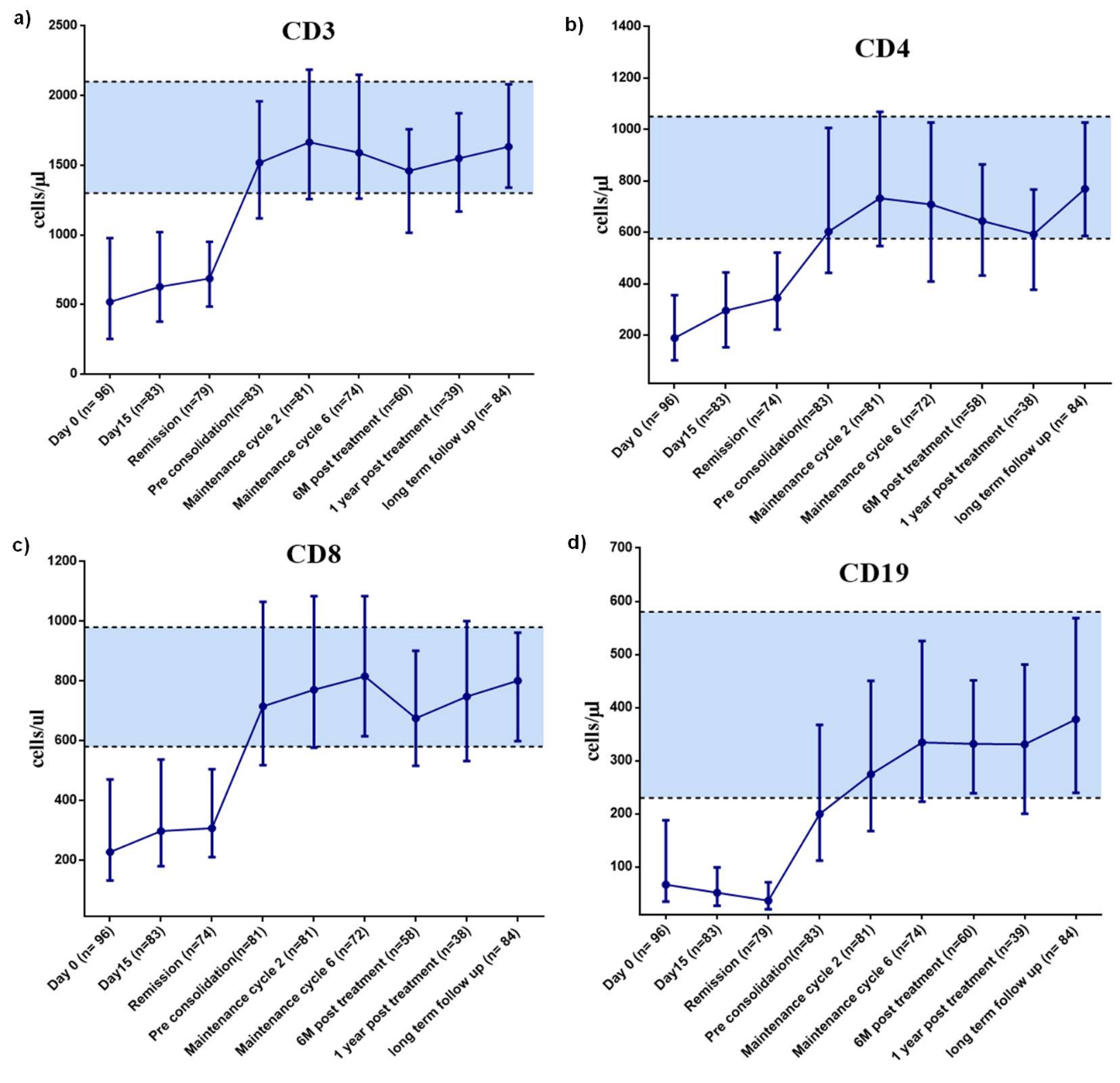
*

**Supplementary figure S6**. The graphs showing the recovery of memory and naive T cells at appropriate time points post treatment with ATO. The error bars indicate median with interquartile ranges. The shaded region represents the 25^th^ to 75^th^ percentile values of long term follow-up samples serve as reference ranges. Number of samples available at each time points were mentioned in brackets. The time points of samples collected were mentioned on x axis Absolute counts are given as cells per microlitre on y axis.

***
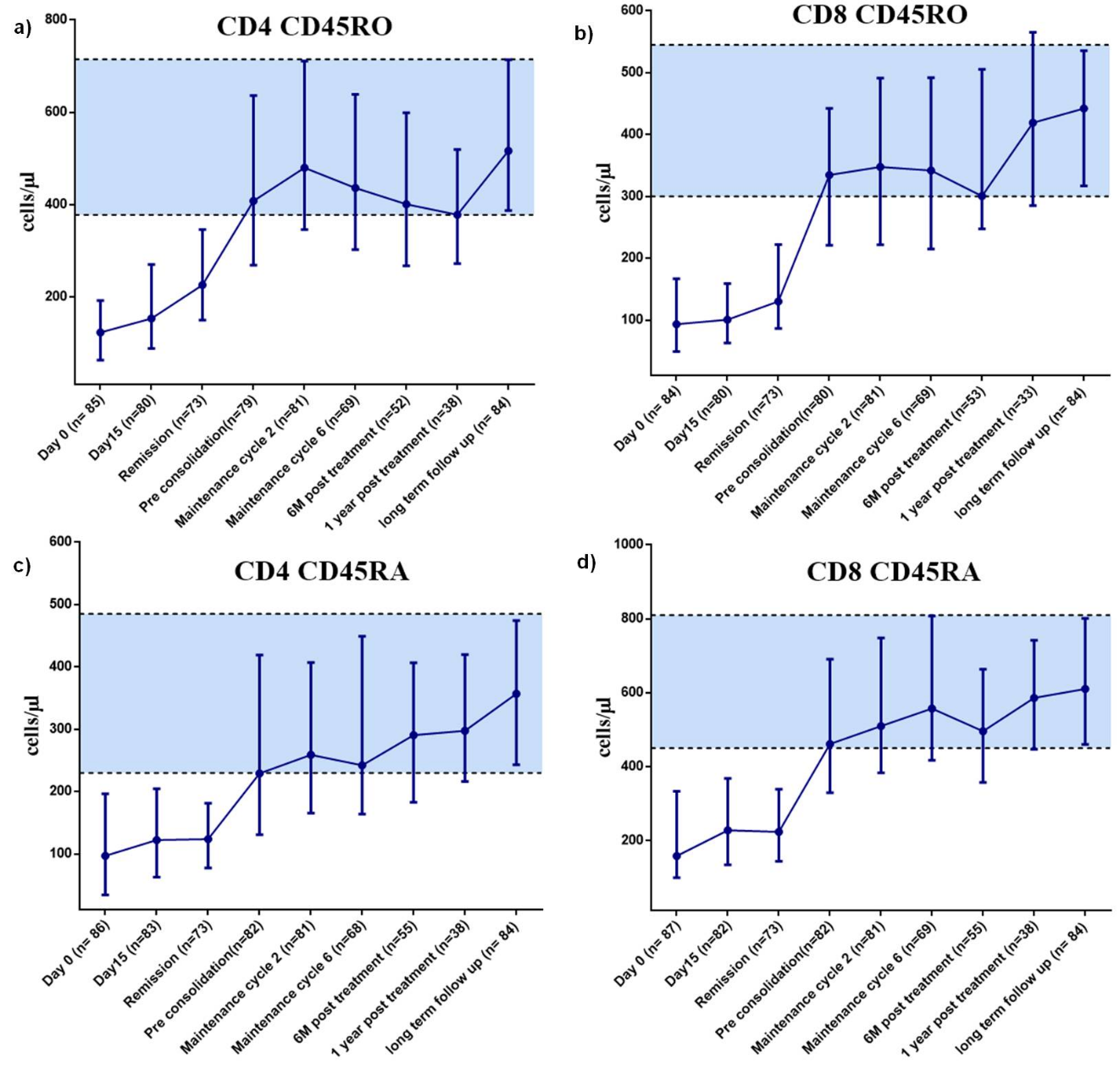
***

**Supplementary figure S7.** The graphs showing the recovery of NK subsets (CD56bright and CD56 dim) at appropriate time points post treatment with ATO. The error bars indicate median with interquartile ranges. The shaded region represents the 25^th^ to 75^th^ percentile values of long term follow-up samples serve as reference ranges. Number of samples available at each time points were mentioned in brackets. The time points of samples collected were mentioned on x axis Absolute counts are given as cells per microlitre on y axis.

**
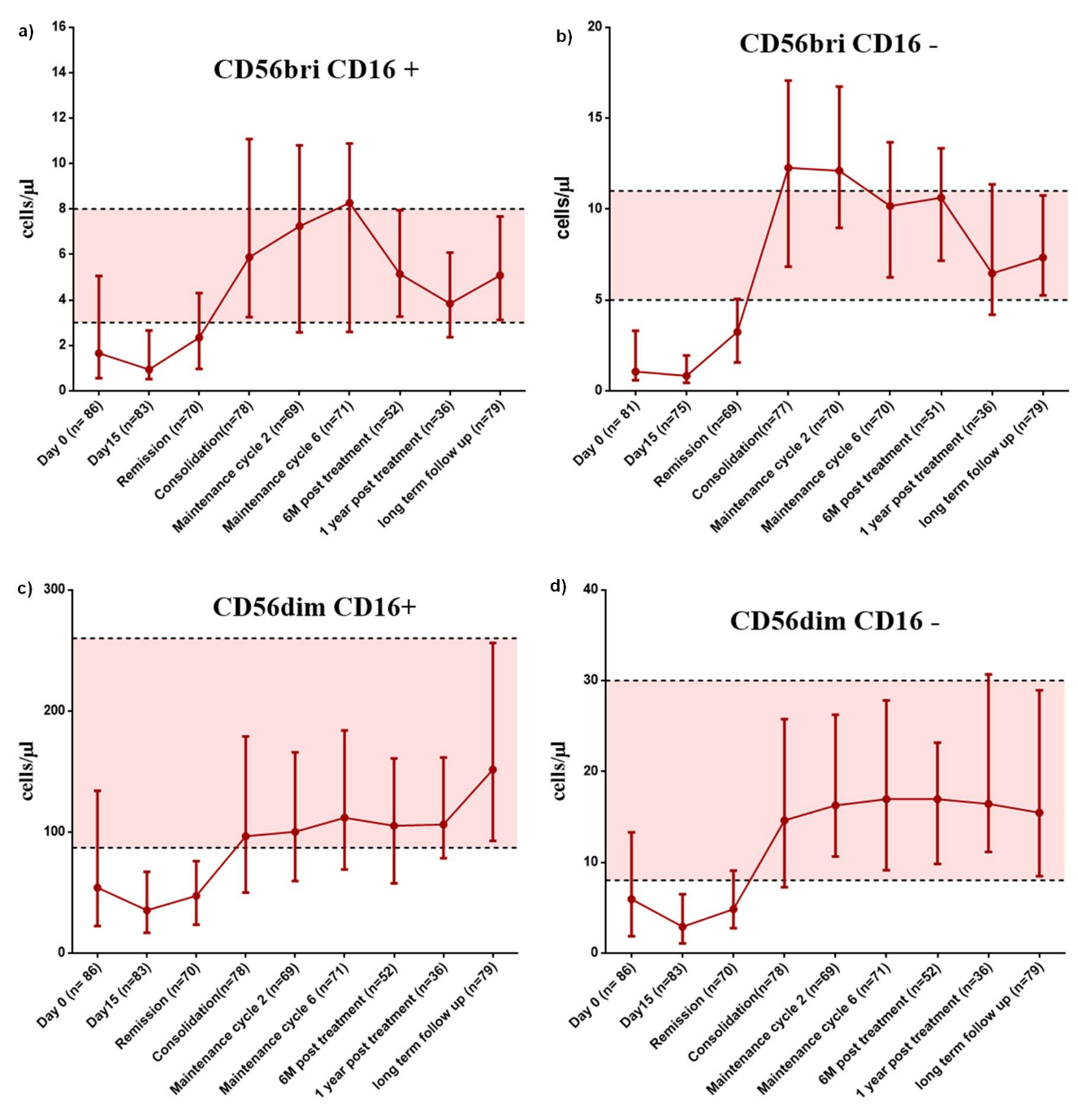
**

**Supplementary Figure S8**. Pie chart showing the percentage of T cells, B cells, NK cells and myeloid differentiated from CD34+ sorted cells with or without 0.5μM ATO on day 14(n=3). 7.7±0.4% cells were positive for CD56^+^CD3^-^ on Day14 in the culture without ATO whereas CD34 seeded in media with ATO had only 5.2±0.4 % on day 14 (n=3). There is a slight increase in the myeloid compartment (81.6±5.5%) on day 14 in culture with ATO when compared with untreated culture (77.8±5.9%). CD3 and CD19 in untreated culture (16.8±3.4% and 1.6±0.7% respectively) was similar to the treated culture (17.4±5.2% and 0.8±0.3% respectively) (n=3) as represented by the pie chart

**
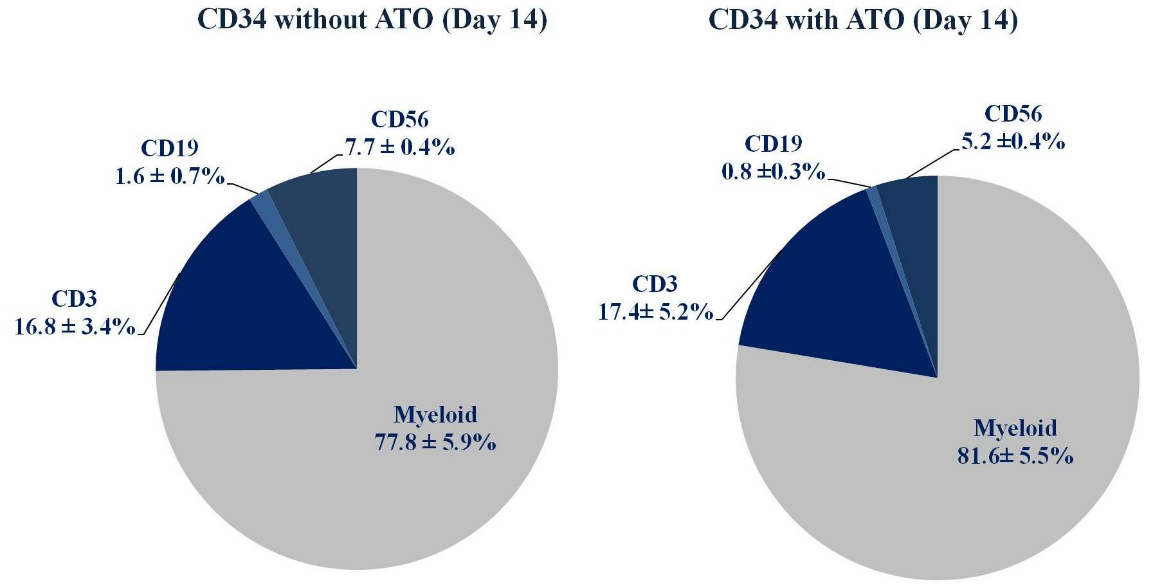
**

**Supplementary Table 1.** Table showing the mean IC 50 values of all the myeloid and lymphoid cell lines treated with ATO in μM concentrations (n=3).

| Cell lines | IC50 (μM)  (mean±SD) (n=3) |
| --- | --- |
| NB4 | 0.94±0.04 |
| NB4-EVAsR1 | 3.13 ± 0.25 |
| UFI | 4.1 ± 0.3 |
| K562 | >6 |
| U937 | 4.03 ± 0.15 |
| HL60 | 2.9 ± 0.1 |
| SUP-B15 | 1.3 ± 0.5 |
| Jurkat E6.1 | >6 |

**Supplementary Table 2.** Table showing the MFI and %NKG2D receptor expression on NK cells (gated on CD56+ve CD3-ve lymphocytes) in APL patients at diagnosis and at remission.

| **PATIENT ID** | **Day 0 (At diagnosis)** | | | | **REMISSION** | | | |
| --- | --- | --- | --- | --- | --- | --- | --- | --- |
|  | **LYMPH GATE**  **( %)** | **GATED ON CD56+CD3- (%)** | **NKG2D**  **(%)** | **NKG2D MFI** | **LYMPH GATE**  **(%)** | **GATED ON CD56+CD3- (%)** | **NKG2D**  **(%)** | **NKG2D**  **MFI** |
| APL1 | 3.39 | 2.3 | 18.7 | 410 | 45.8 | 5.4 | 65.7 | 480 |
| APL2 | 12.6 | 3.5 | 68.5 | 428 | 49 | 9.2 | 85.1 | 457 |
| APL3 | 6.73 | 3.6 | 61.2 | 362 | 21.1 | 4.5 | 90.6 | 525 |
| APL4 | 3.5 | 15 | 32.9 | 224 | 12.3 | 11.5 | 41.7 | 557 |
| APL5 | 2.3 | 2.9 | 28.5 | 259 | 45.2 | 2.3 | 62.5 | 409 |
| APL6 | 6.5 | 2.6 | 77.3 | 427 | 43.9 | 11.4 | 85.5 | 400 |

**Supplementary Table 3.** Univariate analysis of the presence of KIR genes on relapse-free survival.

| **KIR genes** | **relapse (n=16)** | | **non relapse (n=39)** | | **RFS**  **HR(95%CI)** | **p value** |
| --- | --- | --- | --- | --- | --- | --- |
|  | positive | negative | positive | negative |  |  |
| 2DL1 | 16 | 0 | 39 | 0 | - | - |
| 2DL2 | 13 | 3 | 21 | 18 | 3.2(0.91-11.26) | 0.069 |
| 2DL3 | 13 | 3 | 31 | 8 | 1.2(0.34-4.17) | 0.789 |
| 2DL4 | 16 | 0 | 39 | 0 | - | - |
| 2DL5 A/B | 12 | 4 | 28 | 11 | 1.2(0.38-3.66) | 0.775 |
| 2DS1 | 10 | 6 | 23 | 16 | 1.2(0.42-3.19) | 0.774 |
| 2DS2 | 12 | 4 | 20 | 19 | 2.6(0.84-8.06) | 0.098 |
| 2DS3 | 11 | 5 | 16 | 23 | 2.5(0.85-7.06) | 0.097 |
| 2DS4 Del | 6 | 10 | 30 | 9 | 0.8(0.25-2.42) | 0.664 |
| 2DS4 Ins | 6 | 10 | 13 | 26 | 1.2(0.45-3.38) | 0.692 |
| 2DS5 | 13 | 3 | 23 | 16 | 0.9(0.34-2.45) | 0.855 |
| 3DL1 | 13 | 3 | 32 | 7 | 1.0(0.29-3.55) | 0.987 |
| 3DL2 | 16 | 0 | 39 | 0 | - | - |
| 3DL3 | 16 | 0 | 39 | 0 | - | - |
| 3DS1 | 10 | 6 | 22 | 17 | 1.3(0.47-3.53) | 0.631 |
| 2DP1 | 16 | 0 | 39 | 0 | - | - |
| 3DP1 | 16 | 0 | 39 | 0 | - | - |
| Haplotype A/B | 3A and 13B | | 7A and 32B | | 1.0(0.29-3.62) | 0.961 |

**Supplementary table 4**. Baseline clinical and laboratory parameters of newly diagnosed patients with APL.

| **PATIENT CHARACTERISTICS** | |
| --- | --- |
| **Parameters** | **Value n (%) /**  **median (range) (n=96)** |
| Age (years) | 32 (2-59) |
| Sex: male | 47 (48.9%) |
| Hb (g/L) | 79 (26-137) |
| Total WBC (x10^9^/Lt) | 10.6 (0.3-180) |
| Platelet (x10^9^/Lt) | 22 (3-77) |
| LDH (U/L) | 651 (286-2143) |
| Creatinine (mg%) | 0.9 (0.28-1.6) |
| PT (secs) | 13.6 (9.3-26.7) |
| APTT (secs) | 27.2 (23.1-38.3) |
| Fibrinogen (mg/dl) | 163.8 (25-574) |
| Blasts in BM (%) | 86 (48-100) |
| RT PCR bcr1 | 49 (51%) |
| bcr2 | 4 (4.1%) |
| bcr3 | 43 (44.7%) |

**Supplementary table 5.** Table showing the median and range of absolute counts of immune subsets prior to diagnosis, on remission and pre-consolidation. n denotes the number of patients at each time points for each subset

|  | Time points | | | |  | |
| --- | --- | --- | --- | --- | --- | --- |
| Immune subsets | Day0 median(range) | (n) | remission median(range) | (n) | Pre-consolidation median (range) | (n) |
| CD3 | 518.33(27.05-6744.24) | 96 | 686.82(150.26-4961.40) | 79 | 1518.21(323.53-4725.44) | 83 |
| CD19 | 66.63(5.25-2528.69) | 96 | 36.48(0.48-263.93) | 79 | 199.61(10.79-1144.47) | 83 |
| CD56^+^CD3^-^ | 59.53(3.32-756.78) | 96 | 54.6(6.63-383.73) | 78 | 122.49(9.17-547.75) | 83 |
| CD56briCD16^-^ | 1.06(0.06-49.66) | 81 | 3.23(0.07-11.87) | 69 | 12.26(0.49-66.42) | 77 |
| CD56briCD16^+^ | 1.65(0.08-56.07) | 82 | 2.34(0.09-21.44) | 69 | 6.17(0.05-45.36) | 76 |
| CD56bri total | 3.29(0.14-95.52) | 82 | 5.59(0.26-26.14) | 69 | 18.34(0.90-111.77) | 77 |
| CD56dimCD16^+^ | 54.12(4.29-743.75) | 86 | 47.40(2.79-369.36) | 70 | 96.52(7.73-933.07) | 77 |
| CD56dimCD16^-^ | 5.94(0.18-119.72) | 85 | 4.84(0.37-49.47) | 70 | 14.62(0.73-106.91) | 77 |
| CD56^-^CD16^+^ | 4.97(0.14-100.31) | 86 | 6.88(0.63-72.08) | 70 | 20.13(1.40-73.15) | 77 |
| CD56dim total | 58.82(6.87-820.18) | 86 | 52.27(4.72-380.76) | 70 | 118.14(8.46-1039.98) | 77 |
| CD56^+^CD3^+^ | 38.35(1.06-374.68) | 96 | 37.34(2.95-196.98) | 77 | 60.85(13.98-606.50) | 81 |
| CD4CD45RO | 123.59(10.34-1743.42) | 85 | 226.2(48.59-765.73) | 73 | 408.16(58.41-2553.88) | 79 |
| CD8CD45RO | 93.56(6.44-1205.86) | 84 | 130.40(34.99-466.69) | 73 | 334.77(49.71-979.49) | 80 |
| CD4CD45RA | 97.14(3.29-2328.29) | 86 | 123.87(20.16-1809.55) | 73 | 229.14(30.68-2155.54) | 82 |
| CD8CD45RA | 158.71(10.84-2636.81) | 87 | 223.65(24.94-1700.24) | 73 | 461.29(102.03-1672.88) | 82 |
| CD4 | 188.68(8.61-3849.81) | 96 | 344.0(78.57-2485.23) | 74 | 602.57(89.83-3273.29) | 83 |
| CD8 | 226.91(12.65-4363.15) | 96 | 307.04(21.59-1991.92) | 74 | 714.77(113.85-1930) | 81 |
| CD4+CD25+ | 13.79(1.00-168.61) | 90 | 22.22(1.46-87.72) | 68 | 36.24(0.53-176.16) | 76 |
| CD11c+HLADR+Lin- | 16.84(0.21-1962.8) | 96 | 9.4(0.96-77.35) | 75 | 27.72(2.99-161.50) | 73 |
| CD123+HLADR+Lin- | 14.33(0.14-3815.1) | 96 | 2.4(0.48-23.25) | 75 | 3.39(0.75-22.41) | 73 |

**Supplementary table 6.** Table showing the median and range of absolute counts of immune subsets at 6 months post treatment, 1 year post treatment and long term follow-up samples. n denotes the number of patients at each time points for each subset*.*

|  | Time points | | | | |  |
| --- | --- | --- | --- | --- | --- | --- |
| Immune subsets | 6months post treatment median (range) | (n) | 1 year post treatment median (range) | (n) | Long term follow up ( > 2 years) median (range) | (n) |
| CD3 | 1459.24(562.60-3592.41) | 60 | 1550.02 (734.03-2432.80) | 39 | 1633.62(704.92-3462.71) | 84 |
| CD19 | 331.66(89.08-1053.86) | 60 | 330.88 (87.85-755.87) | 39 | 377.79(68.69-1819.71) | 84 |
| CD56+CD3- | 120.84(27.85-484.22) | 60 | 133.95 (43.60-307.53) | 39 | 177.85(61.02-635.37) | 83 |
| CD56briCD16- | 10.62(0.41-41.01) | 51 | 6.87 (1.16-23.13) | 36 | 7.33(1.85-47.77) | 79 |
| CD56briCD16+ | 5.14(0.34-28.95) | 51 | 4.59 (0.44-12.64) | 36 | 5.08(0.42-26.18) | 79 |
| CD56bri | 15.41(1.23-48.95) | 51 | 11.89 (1.75-31.71) | 36 | 12.92(2.78-73.95) | 79 |
| CD56dimCD16+ | 105.21(10.59-438.44) | 52 | 106.16 (34.23-328.54) | 36 | 151.53(26.06-617.27) | 79 |
| CD56dimCD16- | 16.96(3.02-78.33) | 52 | 16.43 ( 4.70-103.35) | 36 | 15.47(2.64-85.82) | 79 |
| CD56-CD16+ | 21.88(7.99-101.33) | 52 | 25.27 (6.29-89.29) | 36 | 25.75(5.57-104.59) | 79 |
| CD56dim | 119.35(27.64-448.88) | 52 | 128.96 (57.92-339.54) | 36 | 167.83(44.57-623.14) | 79 |
| CD56+CD3+ | 56.35(13.09-365.15) | 58 | 63.78 (10.63-234.02) | 39 | 79.56(14.06-243.17) | 83 |
| CD4CD45RO | 401.22(95.06-899.13) | 52 | 378.20 (179.19-839.15) | 33 | 516.52(155.49-1377.53) | 84 |
| CD8CD45RO | 300.59(107-1303.47) | 53 | 419.19 (157.31-763.23) | 33 | 442.21(153.18-970.05) | 84 |
| CD4CD45RA | 290.47(79.95-1573.98) | 55 | 297.66 (126.4-659.81) | 38 | 356.99(130.24-1077.92) | 84 |
| CD8CD45RA | 496.13(164.71-1445.85) | 55 | 585.75 (239.22-1084.56) | 38 | 610.28(258.02-1090.49) | 84 |
| CD4 | 643.86(203.36-1759.67) | 58 | 592.24 (268.79-1320.23) | 38 | 768.87(275.72-2252.58) | 84 |
| CD8 | 674.84(222.29-1822.50) | 58 | 747.73 (372.12-1630.29) | 38 | 800.66(301.83-1753.86) | 84 |
| CD4+CD25+ | 59.58(5.87-231.01) | 56 | 48.03 (12.17-374.48) | 39 | 41.55(10.33-316.59) | 83 |
| CD11c+HLADR+Lin- | 8.64(0.87-56.07) | 53 | 20.9 (4.5-81.6) | 39 | 23.25(6.7-93.6) | 83 |
| CD123+HLADR+Lin- | 1.94(0.54-20.16) | 51 | 4.92 (1.02-20) | 39 | 5.82(1.34-18) | 83 |

**References**

1. Ganesan S, Alex AA, Chendamarai E, Balasundaram N, Palani HK, David S, et al. Rationale and efficacy of proteasome inhibitor combined with arsenic trioxide in the treatment of acute promyelocytic leukemia. Leukemia. 2016 Nov;30(11):2169-78. PubMed PMID: 27560113. Pubmed Central PMCID: PMC5097069. Epub 2016/11/03. eng.

2. Mathews V, George B, Lakshmi KM, Viswabandya A, Bajel A, Balasubramanian P, et al. Single-agent arsenic trioxide in the treatment of newly diagnosed acute promyelocytic leukemia: durable remissions with minimal toxicity. Blood. 2006 Apr 1;107(7):2627-32. PubMed PMID: 16352810. Epub 2005/12/15. Eng.
